# Cardiac Events in Adults Hospitalized for Respiratory Syncytial Virus vs COVID-19 or Influenza

**DOI:** 10.1001/jamanetworkopen.2025.11764

**Published:** 2025-05-22

**Authors:** Liang En Wee, Jue Tao Lim, Reen Wan Li Ho, Calvin J. Chiew, David Chien Boon Lye, Kelvin Bryan Tan

**Affiliations:** 1 National Centre for Infectious Diseases, Singapore; 2 Duke-NUS Graduate Medical School, National University of Singapore, Singapore; 3 Department of Infectious Diseases, Singapore General Hospital, Singapore; 4 Department of Infection Prevention and Epidemiology, Singapore General Hospital, Singapore; 5 Lee Kong Chian School of Medicine, Nanyang Technological University, Singapore; 6 Ministry of Health, Singapore; 7 Department of Infectious Diseases, Tan Tock Seng Hospital, Singapore; 8 Yong Loo Lin School of Medicine, National University of Singapore, Singapore; 9 Saw Swee Hock School of Public Health, National University of Singapore, Singapore

## Abstract

**Importance:**

Respiratory viral infections (RVIs) are associated with elevated cardiovascular risk; however, less is known about cardiac complications after hospitalization for respiratory syncytial virus (RSV) vs other vaccine-preventable RVIs (COVID-19 or influenza).

**Objective:**

To compare the risk of acute cardiovascular complications in adults hospitalized for RSV vs COVID-19 or influenza.

**Design, Setting, and Participants:**

This population-based cross-sectional study, conducted before RSV vaccination rollout in Singapore, assessed all adults hospitalized for RSV or influenza (January 1, 2017, to June 30, 2024) and all adults hospitalized for COVID-19 during Omicron XBB/JN.1 transmission (January 1, 2023, to June 30, 2024).

**Exposure:**

Hospitalization for RSV, influenza (vaccinated or unvaccinated), or COVID-19 (boosted [≥3 vaccine doses] or unboosted [<3 vaccine doses]).

**Main Outcomes and Measures:**

Cardiovascular events during RSV, influenza, or COVID-19 hospitalization, defined as any cardiac, cerebrovascular, or thrombotic event, occurring from admission until discharge or death. Odds of any cardiovascular event (RSV vs COVID-19 or RSV vs influenza) and severe RVI (intensive care unit admission) with or without an acute cardiovascular event were estimated using multivariate logistic regression, adjusted for sociodemographic and clinical characteristics.

**Results:**

A total of 32 960 RVI hospitalizations (mean [SD] patient age, 66.58 [18.99] years; 17 056 [51.7%] female) were included (2148 for RSV, 14 389 for influenza, and 16 423 for COVID-19). Of the 2148 patients hospitalized for RSV, 234 (10.9%) had an acute cardiovascular event. Higher odds of any acute cardiovascular event (adjusted odds ratio [AOR], 1.31; 95% CI, 1.12-1.54) as well as other individual cardiac events were observed in RSV hospitalizations vs boosted COVID-19 (dysrhythmia: AOR, 1.52; 95% CI, 1.19-1.94; heart failure: AOR, 1.75; 95% CI, 1.30-2.35). Similarly, higher odds of any acute cardiovascular event (AOR, 1.58; 95% CI, 1.24-2.01) as well as dysrhythmias or heart failure were observed in patients hospitalized for RSV vs unboosted COVID-19. Odds of a cardiovascular event were not significantly different in RSV vs influenza, except among contemporaneous hospitalizations after the pandemic (2023-2024), where odds of heart failure (AOR, 2.09; 95% CI, 1.21-3.59) were significantly higher in RSV hospitalizations vs vaccine-breakthrough influenza hospitalizations. Occurrence of a cardiovascular event was associated with greater odds of severe RSV requiring intensive care unit admission (AOR, 2.36; 95% CI, 1.21-4.62).

**Conclusions and Relevance:**

In this cross-sectional study, 1 in 10 patients hospitalized for RSV had a concurrent acute cardiovascular event. Odds of cardiac events were significantly higher in RSV vs COVID-19 hospitalizations in both vaccine-boosted and unboosted individuals. In contemporaneous hospitalizations for RSV or influenza after the pandemic (2023-2024), odds of heart failure were significantly higher in RSV hospitalizations vs vaccine-breakthrough influenza hospitalizations. These findings suggest that patients with preexisting cardiovascular risk should consider vaccination against RVIs.

## Key Points

**Question:**

What is the risk of acute cardiovascular complications in adults hospitalized for respiratory syncytial virus (RSV) vs influenza or SARS-CoV-2 Omicron infection?

**Findings:**

In this cross-sectional study comparing 32 960 hospitalizations for RSV with hospitalizations for influenza or Omicron XBB/JN.1 COVID-19, 10.9% of unvaccinated adults hospitalized for RSV had an acute cardiovascular event. Significantly higher odds of cardiac events were observed in patients hospitalized for RSV vs those hospitalized for COVID-19 who had received boosters or those hospitalized for contemporaneous vaccine-breakthrough influenza.

**Meaning:**

Cardiac events were more frequent in unvaccinated patients hospitalized for RSV vs boosted COVID-19 or contemporaneous vaccine-breakthrough influenza; evaluating vaccination’s role in attenuating cardiac risk in patients with respiratory viral infection is important, given availability of RSV vaccines for older adults.

## Teaser

This cross-sectional study assesses the risk of cardiovascular complications in a nationwide cohort of Singaporean adults hospitalized for respiratory syncytial virus, influenza, or COVID-19 during community transmission of Omicron XBB/JN.1 subvariants.

## Introduction

Respiratory viral infections (RVIs) are associated with an elevated risk of cardiovascular complications in the acute phase of infection.^1^ In large, population-based, electronic health record datasets, the risks of acute myocardial infarction (AMI) and cerebrovascular events were substantially elevated for up to a month after RVIs.^1^ Influenza, a common cause of RVI, has been associated with a 4 to 6 times elevated risk of AMI in the immediate week after infection.^2,3^

Less is known, however, about cardiac complications after other RVIs, such as respiratory syncytial virus (RSV), despite RSV accounting for a comparable disease burden to influenza in older adults.^4^ Older adults with preexisting cardiac disease are at increased risk of morbidity due to RSV.^5^ RSV hospitalization can, in turn, be complicated by acute cardiac events, including worsening heart failure, AMI, and arrhythmias, in up to one-fifth of adult patients.^6,7^ Given an increasing body of evidence that highlights potential benefit of vaccination against RVI in improving cardiovascular outcomes,^8^ comparisons between acute cardiac complications of RSV disease and other vaccine-preventable RVIs are important, given recent availability of RSV vaccines for older adults.^9,10^ However, existing comparisons between acute cardiac complications of RSV vs other vaccine-preventable RVIs (eg, influenza and COVID-19) predated the emergence of the milder Omicron SARS-CoV-2 variant and availability of COVID-19 vaccination^11^ or compared RSV and influenza only,^12,13^ limiting generalizability. We assessed the risk of cardiovascular complications in a nationwide study of Singaporean adults hospitalized for RSV, influenza, or COVID-19 during community transmission of Omicron XBB/JN.1 subvariants to better contextualize the burden of acute cardiac events in RSV vs other vaccine-preventable RVIs.

## Methods

### Study Design and Participants

This population-based, cross-sectional study was conducted in Singapore, a Southeast Asian city-state. Year-round RSV transmission has been described in Singapore, given its humid and rainy tropical climate; RSV burden in older adults (aged ≥65 years) is comparable to that in younger children.^14^ Availability of multiplex polymerase chain reaction (PCR) testing for RVIs in hospitalized patients from 2017 onward^15,16^ allowed for surveillance of RSV in hospitalized inpatients, particularly in at-risk populations with preexisting cardiovascular disease.^15^ Yearly influenza vaccination is recommended for older adults, but uptake is low (<15%).^17^ Only RSV or influenza hospitalizations from January 1, 2017, to June 30, 2024 were included, given limited diagnostic testing for RSV before 2017. Because this predated availability of RSV vaccination in Singapore, all RSV hospitalizations were unvaccinated for RSV. Omicron XBB predominated community transmission of COVID-19 in Singapore from 2023 onward^18^; at the end of 2023, JN.1 displaced XBB.^19^ COVID-19 vaccination was first made available in Singapore from December 2020 onward, with booster doses rolled out for older adults in September 2021.^18^ Booster vaccination uptake in older adults was high (≥90%), given public health restrictions imposed on unvaccinated individuals.^20^ Only COVID-19 hospitalizations during XBB/JN.1-predominant transmission from January 1, 2023, to June 30, 2024, were included to provide a more relevant comparison during endemicity. This study was performed as part of national public health research under the Infectious Diseases Act, Singapore; as such, separate ethics review by an institutional review board was not required and informed consent was waived. All data were anonymized before analysis. All results are reported according to Strengthening the Reporting of Observational Studies in Epidemiology (STROBE) guideline.

### Exposure

RSV and influenza hospitalizations among Singaporean adults 18 years or older were identified using RSV- or influenza-specific *International Statistical Classification of Diseases and Related Health Problems, Tenth Revision* (*ICD-10*) codes recorded in Mediclaims, the national health care claims database (RSV: J12.1/J20.5/J21.0/B97.4; influenza: J10.0/ J10.1/ J10.8) (eAppendix in Supplement 1).^21,22^ In Singapore, the national government-administered medical savings scheme (Medisave) and national medical insurance scheme (Medishield) can be claimed against for inpatient care at both public and private health care institutions,^18,19,20^ enabling comprehensive capture of RSV and influenza hospitalizations across different health care settings nationally.^22^ RSV and influenza testing in the hospital setting is incorporated into routine respiratory viral multiplex PCR panels; testing is at physician discretion.^16,22^ For COVID-19, during Omicron XBB–predominant transmission from 2023 onward, testing for hospitalized patients was no longer mandatory and was performed at physicians’ discretion,^18,19^ aligned with the shift toward endemicity. Because the *ICD-10* code (U07.1) was not in use for discharge coding of COVID-19 hospitalizations during the study period, data on COVID-19 hospitalizations were obtained from the national registry maintained by the Ministry of Health, which contained details of all hospitalizations attributed to COVID-19 as well as all COVID-19 test results (PCR or rapid antigen tests) (eAppendix in Supplement 1).^18,19^ Coinfections, defined as concurrent RSV and influenza hospitalizations or an RSV or influenza hospitalization with a concurrent positive SARS-CoV-2 test result, were excluded.

### Outcomes

Cardiovascular events during RSV, influenza, or COVID-19 hospitalization were defined as the composite of any cardiac, cerebrovascular, or thrombotic event that occurred during the period beginning at initial admission and ending at hospital discharge or patient death, whichever was earliest. Any cardiac event was defined as the composite of any dysrhythmia, ischemic heart disease, heart failure, or other cardiac event. Cerebrovascular events included ischemic or hemorrhagic stroke and transient ischemic attack. Thrombotic events included deep venous thrombosis and pulmonary embolism. Major adverse cardiovascular or cerebrovascular events (MACEs) were defined as the first incidence of AMI, stroke, heart failure, ventricular arrhythmia, or cardiac arrest. Events were identified based on *ICD-10* codes recorded in Mediclaims (eAppendix in Supplement 1); previously, this database was used to evaluate the risk of cardiovascular events after COVID-19 hospitalization.^23,24^ We further evaluated whether occurrence of a cardiovascular event was associated with greater severity of RSV, influenza, or COVID-19; severity was defined as an intensive care unit (ICU) admission. COVID-19 hospitalizations were stratified by receipt of booster doses (boosted COVID-19 [≥3 vaccine doses] vs unboosted COVID-19 [<3 vaccine doses]); influenza hospitalizations were stratified by vaccination status, with individuals receiving seasonal influenza vaccination less than 365 days before hospitalization considered vaccinated. Vaccination status for COVID-19 and influenza was determined through linkage with the National Immunization Registry. RSV vaccines were only approved by local regulatory authorities in mid-2024; thus, all RSV hospitalizations included in this study occurred in individuals not previously vaccinated for RSV.

### Covariates

The following covariates were incorporated and defined using information extracted from Ministry of Health databases: demographics (age, sex, and ethnicity, including Chinese, Malay, or other [Indian ethnicity, mixed ethnicity, or other ethnicities, such as Eurasian or Arab]), comorbidity burden (Charlson Comorbidity Index), socioeconomic status, immunocompromised status, and preexisting cardiac history, diabetes, or dyslipidemia. Information on ethnicity was collected to determine whether interethnic differences in risk of cardiovascular events existed, given the multiethnic nature of the Singaporean population. Socioeconomic status was classified by housing type.^20^ Information on medical comorbidities and immunocompromised status was based on diagnosis codes recorded in Mediclaims (eAppendix in Supplement 1).^18,19^

### Statistical Analysis

Descriptive statistics are reported as numbers (percentages) for categorical data, means (SDs) for parametric continuous data, and medians (IQRs) for skewed data. To contrast the burden of acute cardiovascular events in RSV disease compared with COVID-19 and influenza, we report both the unweighted prevalence of cardiovascular events and weighted prevalences; weighting was estimated from generalized, boosted, model-based propensity scores.^25^ Odds of any cardiovascular event (composite and individual events) during hospitalization for RVI (RSV vs COVID-19 and RSV vs influenza) and odds of severe RVI (requiring ICU admission) with and without an acute cardiovascular event were estimated using multivariate logistic regression, adjusted for all available covariates (sociodemographic and clinical characteristics). Age was treated as a continuous variable in the main regression analyses.

The following sensitivity analyses were performed. First, we restricted analyses to adults 60 years or older for whom RSV vaccines are indicated. Second, to account for the possibility that testing patterns for RVI in hospitalized patients may have changed after the COVID-19 pandemic, we compared acute cardiac events across contemporaneous RSV, COVID-19, and influenza hospitalizations (January 1, 2023, to June 30, 2024). Third, only PCR-confirmed COVID-19 cases were included, excluding rapid antigen test–positive cases without corresponding PCR to exclude the possibility that PCR testing might have been differentially used in severe COVID-19 cases. For sensitivity analyses, both unweighted prevalence and weighted prevalence of cardiovascular events, as well as odds of any composite or individual cardiovascular event, were estimated using the same methods as that of the main analysis, with weights recalculated for the subgroup of RVI hospitalizations incorporated in that specific sensitivity analysis. Analyses were conducted using R software, version 4.3.1 (R Foundation for Statistical Computing), and a 95% CI that excluded 1 was taken as the threshold for statistical significance. Correction for multiple comparisons was not made when analyzing each of the separate outcomes in addition to the composite outcome.

## Results

A total of 32 960 Singaporean adults (mean [SD] patient age, 66.58 [18.99] years; 17 056 [51.7%] female and 15 904 [48.3%] male) hospitalized for RSV, influenza, or COVID-19 were included, after excluding coinfections (Figure). A total of 21 009 (63.7%) had at least 1 comorbidity, 6425 (19.5%) had preexisting cardiovascular disease, and 11 593 (35.2%) had diabetes (Table 1). Only 1037 RVI hospitalizations (3.2%) required ICU admission. In total, 2148 adults were hospitalized for RSV and 14 389 adults were hospitalized for influenza from January 1, 2017, to June 30, 2024; 16 423 adults were hospitalized for COVID-19 in 2023 to 2024 during Omicron XBB/JN.1–predominant transmission (Figure).

**Figure.**
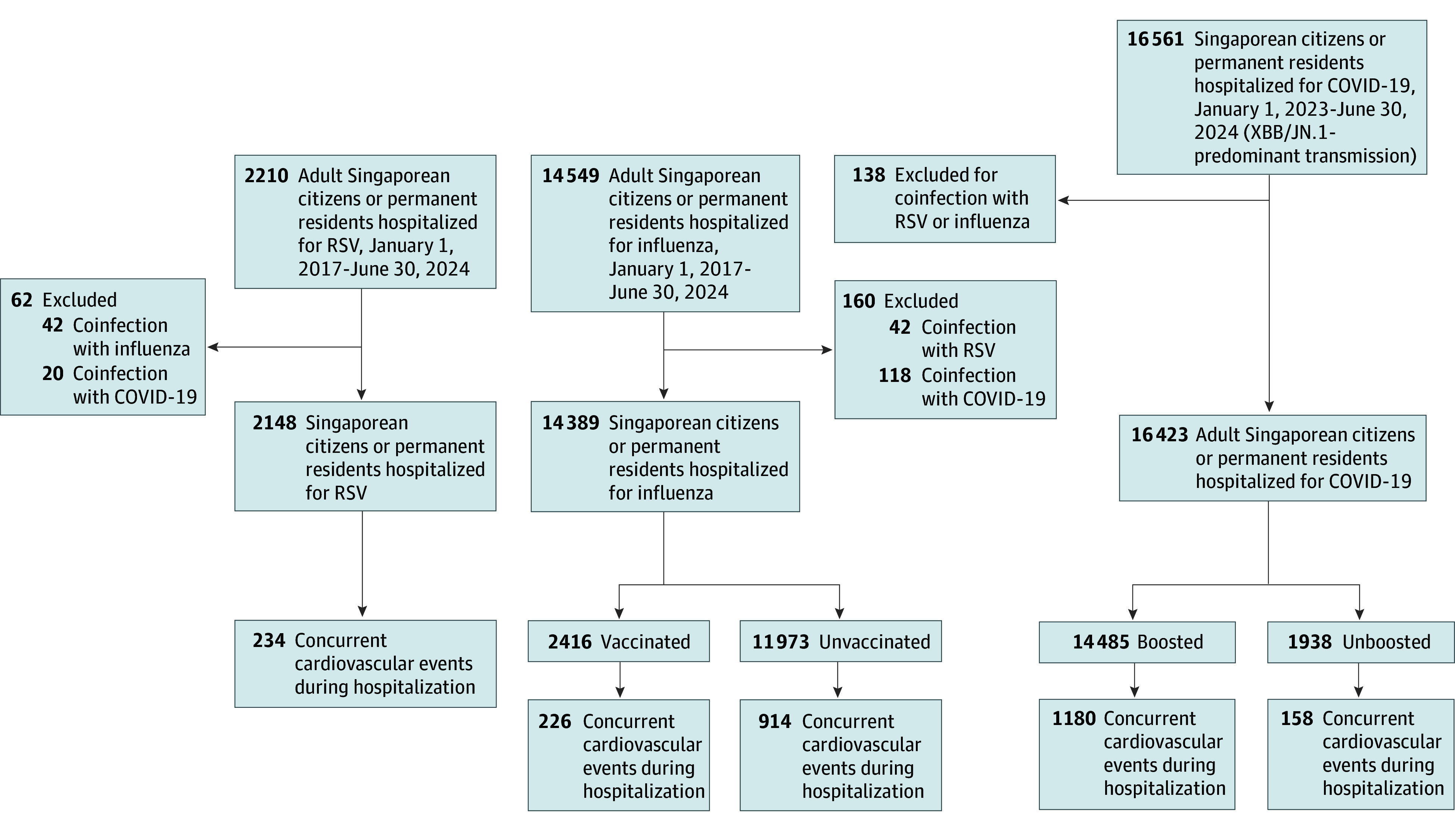
Adult Hospitalizations for Respiratory Syncytial Virus (RSV), Influenza, and COVID-19 With Number of Hospitalizations That Had Concurrent Cardiovascular Events

**Table 1.** Sociodemographic and Clinical Characteristics of Hospitalizations for RSV, Influenza, and Omicron XBB/JN.1 SARS-CoV-2 in Singaporean Adults

Sociodemographic or clinical characteristic	No. (%)^a^				
	RSV hospitalizations, 2017-2024	Influenza hospitalizations, 2017-2024^b^		Omicron XBB/JN.1 COVID-19 hospitalizations, 2023-2024^c^	
		Vaccinated	Unvaccinated	Boosted	Unboosted
Total No. of hospitalizations	2148 (6.5)	2416 (7.3)	11 973 (36.3)	14 485 (43.9)	1938 (5.9)
Age, mean (SD), y	70.6 (16.8)	68.0 (17.4)	58.2 (20.2)	71.8 (16.1)	73.3 (16.9)
Age group, y					
<60	454 (21.1)	531 (22.0)	5583 (46.6)	2595 (17.9)	354 (18.3)
60-69	421 (19.6)	488 (20.2)	2254 (18.8)	2550 (17.6)	301 (15.5)
70-79	528 (24.6)	782 (32.4)	2258 (18.9)	4083 (28.2)	453 (23.4)
≥80	745 (34.7)	615 (25.5)	1878 (15.7)	5257 (36.3)	830 (42.8)
Ethnicity					
Chinese	1323 (61.6)	1482 (61.3)	7006 (58.5)	11 042 (76.2)	1432 (73.9)
Malay	493 (23.0)	600 (24.8)	2927 (24.4)	2095 (14.5)	247 (12.7)
Other^d^	332 (15.5)	334 (13.8)	2040 (17.0)	1348 (9.3)	259 (13.4)
Gender					
Female	1281 (59.6)	1194 (49.4)	6534 (54.6)	7034 (48.6)	1013 (52.3)
Male	867 (40.4)	1222 (50.6)	5259 (43.9)	7454 (51.5)	925 (47.7)
Socioeconomic status (housing type)^e^					
Public, 1-2 room(s)	256 (11.9)	287 (11.9)	1328 (11.1)	1607 (11.1)	254 (13.1)
Public, 3 rooms	464 (21.6)	500 (20.7)	2354 (19.7)	3253 (22.5)	435 (22.4)
Public, 4 rooms	668 (31.1)	808 (33.4)	3706 (31.0)	4476 (30.9)	619 (31.9)
Public, 5 rooms	596 (27.7)	646 (26.7)	3735 (31.2)	3988 (27.5)	472 (24.4)
Private housing	164 (7.6)	175 (7.2)	850 (7.1)	1161 (8.0)	158 (8.2)
Comorbidity burden^f^					
Charlson Comorbidity Index of 0	524 (24.4)	615 (25.5)	6310 (52.7)	4010 (27.7)	492 (25.4)
Charlson Comorbidity Index of 1-2 (mild)	1187 (55.3)	1387 (57.4)	4654 (38.9)	7425 (51.3)	944 (48.7)
Charlson Comorbidity Index ≥3 (moderate-severe)	437 (20.3)	414 (17.1)	1009 (8.4)	3050 (21.1)	502 (25.9)
Immunocompromised^g^	466 (21.7)	484 (20.0)	1419 (11.9)	2715 (18.7)	424 (21.9)
Preexisting cardiac history^h^	568 (26.4)	509 (21.1)	1454 (12.1)	3328 (23.0)	566 (29.2)
Preexisting dyslipidemia	135 (6.3)	143 (5.9)	404 (3.4)	883 (6.1)	132 (6.8)
Preexisting diabetes	831 (38.7)	954 (39.5)	3213 (26.8)	5845 (40.4)	750 (38.7)
Preexisting chronic lung disease	528 (24.6)	704 (29.1)	1308 (10.9)	1743 (12.0)	242 (12.5)
Clinical outcomes and health care uyse					
Required ICU admission	87 (4.1)	84 (3.5)	445 (3.7)	362 (2.5)	59 (3.0)
Length of stay, median (IQR), d	4.0 (2.0-8.0)	4.0 (2.0-6.0)	3.0 (2.0-5.0)	4.0 (2.0-8.0)	5.0 (2.0-10.0)
ICU length of stay, median (IQR), d	3.0 (2.0-5.0)	2.5 (1.0-5.0)	3.0 (1.0-6.0)	2.0 (1.0-5.0)	3.0 (1.0-10.0)

Abbreviations: ICU, intensive care unit; RSV, respiratory syncytial virus.

^a^ Unless otherwise indicated.

^b^ Individuals who had received seasonal influenza vaccination less than 365 days before hospitalization were considered vaccinated.

^c^ During 2023 to 2024, community transmission of COVID-19 was dominated by various Omicron XBB subvariants and subsequently JN.1. Boosting was defined as having received at least a third messenger RNA vaccine dose 6 months or more after completion of an initial 2-dose messenger RNA primary vaccine series.

^d^ Including individuals of Indian ethnicity, mixed ethnicity, or other ethnicities (eg, Eurasian and Arab); categories were consolidated because individual numbers were too small for separate analysis.

^e^ Housing type was used as an indicator of socioeconomic status.

^f^ Comorbidity burden was defined using the Charlson Comorbidity Index, which consists of the following comorbidities: myocardial infarction, chronic heart failure, peripheral vascular disease, cerebrovascular accident, dementia, chronic obstructive pulmonary disease, connective tissue disease, peptic ulcer disease, diabetes, hemiplegia, liver disease, moderate to severe renal impairment, solid tumor, leukemia, and HIV infection with AIDS.

^g^ Immunocompromised status was defined as presence of solid malignant neoplasm, hematologic malignant neoplasm, rheumatologic or inflammatory disorders, organ or stem cell transplant, or other intrinsic immune condition or immunodeficiency.

^h^ Preexisting cardiac history was defined as history of ischemic heart disease or heart failure.

A total of 234 RSV hospitalizations (10.9%) were associated with an acute cardiovascular event; 220 (94.1%) involved cardiac events (99 dysrhythmias, 66 heart failure episodes, and 61 ischemic heart disease episodes). Atrial fibrillation or flutter was the most common dysrhythmia, occurring in 60 of 99 patients (60.6%). Preexisting cardiac history was associated with higher odds of an acute cardiovascular event (adjusted odds ratio [AOR], 2.53; 95% CI, 1.84-3.48) among RSV hospitalizations (eTable 1 in Supplement 1) and among influenza and COVID-19 hospitalizations (eTables 2 and 3 in Supplement 1). Higher odds of any acute cardiovascular event (AOR, 1.31; 95% CI, 1.12-1.54) were observed in RSV hospitalizations vs COVID-19 hospitalizations in vaccine-boosted individuals; similarly, higher odds of acute cardiovascular events (AOR, 1.58; 95% CI, 1.24-2.01) were observed in RSV hospitalizations vs COVID-19 hospitalizations in unboosted individuals. Odds of any cardiovascular event were not significantly different in RSV hospitalizations vs influenza hospitalizations stratified by vaccination status (Table 2).

**Table 2.** Prevalence of Acute Cardiovascular Events (Composite) Among Hospitalization for RSV, Influenza, and Omicron XBB/JN.1 SARS-CoV-2 in Singaporean Adults

RVI	Any cardiovascular event^a^				Any cardiac event^b^				Any MACE^c^			
	RSV, No. (%)	Other RVI, No. (%) (reference)		AOR^e^ (95% CI)	RSV, No. (%)	Other RVI, No. (%) (reference)		AOR^e^ (95% CI)	RSV, No. (%)	Other RVI, No. (%) (reference)		AOR^e^ (95% CI)
		Unweighted	Weighted^d^			Unweighted	Weighted^d^			Unweighted	Weighted^d^	
RSV vs COVID-19 (reference), boosted^f^	234 (10.9)	1180 (8.1)	177 (8.4)	1.31 (1.12-1.54)	220 (10.2)	941 (6.5)	146 (7.0)	1.52 (1.28-1.79)	113 (5.3)	681 (4.7)	102 (4.8)	1.05 (0.84-1.31)
RSV vs COVID-19 (reference), unboosted^f^	234 (10.9)	158 (8.2)	142 (7.2)	1.58 (1.24-2.01)	220 (10.2)	131 (6.8)	111 (5.6)	1.93 (1.49-2.50)	113 (5.3)	92 (4.7)	77 (3.9)	1.33 (0.97-1.82)
RSV vs unvaccinated influenza (reference)^g^	234 (10.9)	914 (7.6)	223 (10.6)	1.03 (0.87-1.21)	220 (10.2)	821 (6.9)	203 (9.6)	1.06 (0.89-1.26)	113 (5.3)	421 (3.5)	115 (5.5)	0.95 (0.75-1.19)
RSV vs vaccinated influenza (reference)^g^	234 (10.9)	226 (9.4)	226 (11.2)	0.95 (0.77-1.18)	220 (10.2)	206 (8.5)	207 (10.2)	0.98 (0.78-1.23)	113 (5.3)	101 (4.2)	103 (5.1)	1.01 (0.74-1.37)

Abbreviations: AOR, adjusted odds ratio; MACE, major adverse cardiovascular event; RSV, respiratory syncytial virus; RVI, respiratory viral infection.

^a^ Any cardiovascular event was defined as the composite of any cardiac, cerebrovascular, or thrombotic event.

^b^ Any cardiac event was defined as the composite of any dysrhythmia, ischemic heart disease, heart failure, or other cardiac event.

^c^ MACE was defined as the first incidence of myocardial infarction, stroke, heart failure, ventricular arrhythmia, or sudden cardiovascular collapse.

^d^ Weighted prevalence of cardiovascular events, calculated using propensity score weighting for the following covariates: age, gender, ethnicity, socioeconomic status, comorbidity burden (Charlson Comorbidity Index), immunocompromised status, preexisting cardiac history, preexisting diabetes, and preexisting dyslipidemia, with RSV hospitalization as the reference category.

^e^ AOR estimated using multivariate logistic regression models, adjusting for age, gender, ethnicity, socioeconomic status, comorbidity burden (Charlson Comorbidity Index), immunocompromised status, preexisting cardiac history, preexisting diabetes, preexisting dyslipidemia, and intensive care unit admission during hospitalization for RVI. Age was treated as a continuous variable in regression analyses.

^f^ RSV hospitalizations were compared against hospitalizations attributed to COVID-19 in January 2023 to June 2024 (COVID-19 as the reference category); during 2023 to 2024, community transmission of COVID-19 was dominated by various Omicron XBB subvariants and subsequently JN.1. Boosting was defined as having received at least a third messenger RNA vaccine dose 6 months or more after completion of an initial 2-dose messenger RNA primary vaccine series.

^g^ RSV hospitalizations were compared against hospitalization attributed to influenza from January 2017 to June 2024 (influenza hospitalization as the reference category). Individuals who had received seasonal influenza vaccination less than 365 days before hospitalization were considered vaccinated.

For individual cardiovascular events, higher odds of dysrhythmia (AOR, 1.52; 95% CI, 1.19-1.94), heart failure (AOR, 1.75; 95% CI, 1.30-2.35), and other cardiac events, including cardiac arrest, nonischemic cardiomyopathy, myocarditis, and pericarditis (AOR, 1.63; 95% CI, 1.23-2.17) were observed in RSV hospitalizations vs COVID-19 hospitalizations in vaccine-boosted individuals. Similarly, higher odds of dysrhythmia (AOR, 1.59; 95% CI, 1.09-2.30), heart failure (AOR, 2.92; 95% CI, 1.83-4.66), and other cardiac events (AOR, 2.40; 95% CI, 1.54-3.75) were observed in RSV hospitalizations vs COVID-19 hospitalizations in unboosted individuals (Table 3). However, lower odds of cerebrovascular events were observed in RSV hospitalizations vs vaccine-boosted COVID-19 hospitalizations (AOR, 0.28; 95% CI, 0.14-0.56). Odds of an individual cardiovascular event were not significantly different in RSV hospitalizations vs influenza hospitalizations stratified by vaccination status (Table 3). Occurrence of any cardiovascular event (AOR, 2.36; 95% CI, 1.21-4.62), any cardiac event (AOR, 2.54; 95% CI, 1.29-4.98), any MACE (AOR, 3.15; 95% CI, 1.40-7.10), and any ischemic heart disease episode (AOR, 4.90; 95% CI, 1.81-13.25) in an RSV hospitalization was associated with greater odds of severe RSV requiring ICU admission (Table 4). Similarly, occurrence of cardiovascular events in influenza and COVID-19 hospitalizations was associated with greater odds of requiring ICU admission.

**Table 3.** Prevalence of Acute Cardiovascular Events (Individual) Among Hospitalizations for RSV, Influenza, and Omicron XBB/JN.1 SARS-CoV-2 in Singaporean Adults

Acute cardiovascular event	RSV, No. (%)	Boosted COVID-19 group, No. (%)		AOR (95% CI) for RSV vs boosted COVID-19 groups^c^	Unboosted COVID-19		AOR (95% CI) for RSV vs unboosted COVID-19^c^	Vaccinated influenza		AOR (95% CI) for RSV vs vaccinated influenza^c^	Unvaccinated influenza		AOR (95% CI) for RSV vs unvaccinated influenza^c^
		Unweighted	Weighted^a^^,^^b^		Unweighted	Weighted^a^^,^^b^		Unweighted	Weighted^a^^,^^d^		Unweighted	Weighted^a^^,^^d^	
Any dysrhythmia^e^	99 (4.6)	396 (2.7)	65 (3.1)	1.52 (1.19-1.94)	61 (3.1)	59 (3.0)	1.59 (1.09-2.30)	105 (4.3)	113 (5.6)	0.80 (0.59-1.10)	420 (3.5)	100 (4.7)	0.97 (0.76-1.24)
Any ischemic heart disease	61 (2.8)	405 (2.8)	58 (2.8)	0.99 (0.74-1.31)	48 (2.5)	36 (1.8)	1.53 (0.99-2.33)	70 (2.9)	62 (3.1)	0.88 (0.61-1.29)	255 (2.1)	64 (3.0)	0.91 (0.67-1.24)
Any heart failure	66 (3.1)	223 (1.5)	37 (1.8)	1.75 (1.30-2.35)	30 (1.5)	21 (1.1)	2.92 (1.83-4.66)	40 (1.7)	45 (2.2)	1.38 (0.88-2.17)	185 (1.5)	58 (2.8)	1.11 (0.81-1.51)
Any other cardiac event^f^	72 (3.4)	262 (1.8)	43 (2.0)	1.63 (1.23-2.17)	38 (2.0)	28 (1.4)	2.40 (1.54-3.75)	47 (1.9)	51 (2.5)	1.33 (0.87-2.03)	203 (1.7)	60 (2.8)	1.18 (0.87-1.59)
Any thrombotic event	7 (0.3)	73 (0.5)	10 (0.5)	0.67 (0.30-1.49)	12 (0.6)	16 (0.8)	0.38 (0.14-1.06)	9 (0.4)	8 (0.4)	0.79 (0.28-2.23)	35 (0.3)	7 (0.3)	1.03 (0.44-2.42)
Any cerebrovascular event	9 (0.4)	199 (1.4)	30 (1.4)	0.28 (0.14-0.56)	NA	NA	NA	NA	NA	NA	67 (0.6)	15 (0.7)	0.60 (0.29-1.25)

Abbreviations: AOR, adjusted odds ratio; NA, not applicable; RSV, respiratory syncytial virus.

^a^ Weighted prevalence of cardiovascular events was calculated using propensity score weighting for the following covariates: age, gender, ethnicity, socioeconomic status, comorbidity burden (Charlson Comorbidity Index), immunocompromised status, preexisting cardiac history, preexisting diabetes, and preexisting dyslipidemia, with RSV hospitalization as the reference category.

^b^ Hospitalization attributed to RSV from January 2017 to June 2024 was compared against hospitalization attributed to COVID-19 from January 2023 to June 2024 (COVID-19 as the reference category); during 2023 to 2024, community transmission of COVID-19 was dominated by various Omicron XBB subvariants and subsequently JN.1. Boosting was defined as having received at least a third messenger RNA vaccine dose 6 months or more after completion of an initial 2-dose messenger RNA primary vaccine series.

^c^ AORs were estimated using multivariate logistic regression models, adjusting for age, gender, ethnicity, socioeconomic status, comorbidity burden (Charlson Comorbidity Index), immunocompromised status, preexisting cardiac history, preexisting diabetes, preexisting dyslipidemia, and intensive care unit admission during hospitalization for respiratory viral infection. Age was treated as a continuous variable in regression analyses.

^d^ RSV hospitalization was compared against hospitalization attributed to influenza from January 2017 to June 2024 (influenza hospitalization as the reference category). Individuals who had received seasonal influenza vaccination less than 365 days before hospitalization were considered vaccinated.

^e^ Among dysrhythmias recorded during RSV hospitalization (n = 99), the most common dysrhythmia was atrial fibrillation/flutter (n = 60), followed by tachycardia, unspecified (n = 20) and supraventricular tachycardia (n = 8). Among dysrhythmias recorded during COVID-19 hospitalization (n = 457), the most common dysrhythmia was atrial fibrillation or flutter (n = 271), followed by tachycardia, unspecified (n = 59), supraventricular tachycardia (n = 30), and bradycardia, unspecified (n = 30). Among dysrhythmias recorded during influenza hospitalization (n = 525), the most common dysrhythmia was atrial fibrillation or flutter (n = 280), followed by tachycardia, unspecified (n = 123) and supraventricular tachycardia (n = 31).

^f^ Any other cardiac event included nonischemic cardiomyopathy, cardiac arrest, cardiogenic shock, and inflammatory heart diseases (myocarditis or pericarditis).

**Table 4.** Odds of ICU Admission Stratified by the Presence of an Acute Cardiovascular Event During Short-Term Hospitalizations for RSV, Influenza, and Omicron XBB/JN.1 SARS-CoV-2 in Singaporean Adults

Hospitalization cause	ICU admission, No. (%)^a^		AOR (95% CI)^d^	ICU admission, No. (%)^b^		AOR (95% CI)^d^	ICU admission, No. (%)^c^		AOR (95% CI)^d^	ICU admission, No. (%)			AOR (95% CI)^d^	ICU admission, No. (%)		AOR (95% CI)^d^
	Any CV event	No CV event (reference)		Any cardiac event	No cardiac event (reference)		Any MAC^c^	No MACE (reference)		Any IHD event	No IHD event (reference)			Any HF event	No HF event	
RSV (2017-2024)	12 (8.3)	75 (3.7)	2.36 (1.21-4.62)	12 (8.7)	75 (3.7)	2.54 (1.29-4.98)	8 (10.7)	79 (3.8)	3.15 (1.40-7.10)	6 (17.1)	81 (3.8)	4.90 (1.81-13.25)		4 (8.7)	83 (3.9)	2.52 (0.88-7.23)
Unvaccinated influenza (2017-2024)^e^	78 (11.9)	367 (3.2)	3.60 (2.50-5.18)	71 (12.0)	374 (3.3)	3.48 (2.38-5.07)	52 (16.7)	393 (3.4)	5.25 (3.46-7.97)	32 (18.4)	413 (3.5)	4.86 (2.97-7.96)		21 (15.7)	424 (3.6)	4.04 (2.18-7.48)
Vaccinated influenza (2017-2024)^e^	15 (10.6)	69 (3.0)	3.63 (1.81-7.28)	15 (11.5)	69 (3.0)	4.01 (1.99-8.07)	12 (18.5)	72 (3.1)	6.48 (2.94-14.30)	8 (16.7)	76 (3.2)	7.49 (3.01-18.61)		3 (14.3)	81 (3.4)	2.49 (0.56-11.06)
Boosted Omicron XBB/JN.1 COVID-19 (2023-2024)^f^	106 (11.3)	256 (1.9)	6.44 (4.83-8.59)	94 (12.6)	268 (2.0)	7.16 (5.27-9.74)	72 (13.3)	290 (2.1)	7.17 (5.18-9.93)	53 (16.5)	309 (2.2)	7.61 (5.14-11.26)		20 (12.0)	342 (2.4)	5.09 (2.92-8.90)
Unboosted Omicron XBB/JN.1 COVID-19 (2023-2024) ^f^	15 (12.0)	44 (2.4)	7.07 (3.40-14.69)	12 (11.7)	47 (2.6)	5.55 (2.52-12.23)	13 (17.8)	46 (2.5)	15.13 (6.99-32.77)	5 (13.5)	54 (2.8)	4.39 (1.37-14.07)		3 (15.0)	56 (2.9)	7.83 (1.56-39.42)

Abbreviations: AOR, adjusted odds ratio; CV, cardiovascular; HF, heart failure; ICU, intensive care unit; IHD, ischemic heart disease; MACE, major adverse cardiovascular event; RSV, respiratory syncytial virus.

^a^ Any cardiovascular event was defined as the composite of any cardiac, cerebrovascular, or thrombotic event.

^b^ Any cardiac event was defined as the composite of any dysrhythmia, ischemic heart disease, heart failure, or other cardiac event.

^c^ MACE was defined as the first incidence of myocardial infarction, stroke, heart failure, ventricular arrhythmia, or sudden cardiovascular collapse.

^d^ AORs were estimated using multivariate logistic regression models, adjusting for age, gender, ethnicity, socioeconomic status, comorbidity burden (Charlson Comorbidity Index), immunocompromised status, preexisting cardiac history, preexisting diabetes, preexisting dyslipidemia, and ICU admission during hospitalization for respiratory viral infection. Age was treated as a continuous variable in regression analyses.

^e^ Individuals who had received seasonal influenza vaccination less than 365 days before hospitalization were considered vaccinated.

^f^ Boosting was defined as having received at least a third messenger RNA vaccine dose 6 months or more after completion of an initial 2-dose messenger RNA primary vaccine series.

In sensitivity analyses, when the population was restricted to older adults 60 years or older, comparable results were found. Demographic characteristics of RVI hospitalizations in older adults are described in eTable 4 in Supplement 1. Higher odds of composite and individual cardiovascular events were observed in RSV hospitalizations vs COVID-19 in older adults (eTables 5 and 6 in Supplement 1), with increased odds of ICU admission among RVI hospitalizations with a cardiovascular event (eTable 7 in Supplement 1). Odds of any cardiovascular event were not significantly different between RSV and influenza hospitalizations in older adults stratified by vaccination status (eTable 5 in Supplement 1). Similarly, when restricted to contemporaneous RVI hospitalizations (January 1, 2023, to June 30, 2024) in the postpandemic period (eTable 8 in Supplement 1), higher odds of composite and individual cardiovascular events were observed in RSV hospitalizations vs COVID-19 (eTables 9 and 10 in Supplement 1). Although odds of any cardiovascular event (composite) were not significantly different between RSV and unvaccinated influenza, odds of heart failure and other cardiac events were significantly higher in adults hospitalized after the pandemic (2023-2024) for RSV vs vaccine-breakthrough influenza (heart failure: AOR, 2.09; 95% CI, 1.21-3.59; other cardiac events: AOR, 1.94; 95% CI, 1.18-3.19) (eTable 10 in Supplement 1). Odds of ICU admission were increased among contemporaneous RVI hospitalizations with a cardiovascular event (eTable 11 in Supplement 1). When restricted to PCR-confirmed COVID-19 hospitalizations, higher odds of composite and individual cardiovascular events were still observed in RSV hospitalizations vs COVID-19 (eTables 12 and 13 in Supplement 1).

## Discussion

In this large population-based study of hospitalizations for RSV, influenza, and COVID-19 (N = 32 960), 1 in 10 RSV hospitalizations had an acute cardiovascular event. Occurrence of any cardiovascular event or MACE was associated with greater odds of severe RVI requiring ICU admission. Higher odds of any acute cardiovascular event, dysrhythmia, heart failure, and other cardiac events but lower odds of cerebrovascular events were observed in RSV vs boosted and unboosted COVID-19 hospitalizations. Although odds of an individual cardiovascular event were not significantly different overall in RSV hospitalizations vs influenza hospitalizations stratified by vaccination status, odds of heart failure and other cardiac events were significantly higher in older adults hospitalized after the pandemic (2023-2024) for RSV vs vaccine-breakthrough influenza.

Hospitalization for RSV can be complicated by acute cardiac events. In large cross-sectional studies of hospitalized adults with RVI, an acute cardiac event was experienced in one-quarter of RSV hospitalizations^7^ but in only 10% of COVID-19 hospitalizations in the pre-Omicron era.^26^ However, the burden of acute cardiac events in RSV vs COVID-19 was not directly compared. Furthermore, restriction to the pre-Omicron period limited generalizability,^26^ given milder Omicron infection and subsequent availability of booster vaccination. In a multicenter US study^27^ conducted during Omicron-predominant transmission, although RSV disease had comparable clinical severity to that of influenza and COVID-19, the prevalence of acute cardiac events was not directly compared. RSV hospitalizations were associated with greater odds of cardiac events compared with both vaccine-boosted and unboosted COVID-19 hospitalizations in the Omicron era. This finding likely reflects milder severity of Omicron-variant infection as well as the high rate of COVID-19 vaccination uptake (≥90%) in our population, contributing to greater clinical severity of RSV vis-à-vis COVID-19 and thus higher risk of cardiac events. We found no significant difference in acute cardiac events between RSV and influenza hospitalizations, except in contemporaneous RSV and influenza hospitalizations after the pandemic (2023-2024), in which odds of heart failure and other cardiac events were significantly higher in RSV hospitalizations vs vaccine-breakthrough influenza but not unvaccinated influenza. This finding likely reflects the impact of influenza vaccination in mitigating severe disease during the rebound in seasonal influenza that occurred after the pandemic.^28^

Cardiac manifestations of RSV are hypothesized to arise directly from myocardial injury or indirectly via a postinflammatory response and increased cardiovascular strain attributed to pulmonary disease.^5^ Dysrhythmia was the most frequently encountered cardiac event after RSV hospitalization, with atrial fibrillation most commonly reported. Although few data exist on cardiac events after RSV infection in older adults, cardiovascular complications have been reported after pediatric RSV infection.^29,30^ In a cohort of pediatric hospitalizations for bronchiolitis that were tested for RSV and systematically evaluated for cardiac events via 24-hour Holter monitoring and echocardiography, arrhythmias were identified in 76.5% of RSV-positive patients but in less than 5% of RSV-negative cases.^30^ Additional studies are needed to clarify pathophysiologic mechanisms underlying dysrhythmia, symptomatic correlation, and long-term resolution in RSV-infected adults. Increased risk of cardiac events but lower risk of cerebrovascular events in RSV hospitalizations vs COVID-19 may reflect pathogen-specific differences in extrapulmonary manifestations and tropism^31^; COVID-19 is associated with increased long-term risk of cardiac and neurologic manifestations in the postacute phase of infection.^24,32,33^

Occurrence of any cardiovascular event or MACE was associated with greater odds of severe RSV requiring ICU admission. In a large cross-sectional study across a US hospital network, the already high in-hospital mortality rate of 5% for older adults with RSV-associated hospitalizations was doubled among those who experienced a concurrent acute cardiac event.^7^ In our population, 4% of RSV hospitalizations required ICU admission; odds of ICU admission were similarly doubled among those with a concurrent cardiovascular event. Individuals with preexisting cardiac conditions had higher odds of a concurrent acute cardiac event during RSV hospitalization. In a modeling study^34^ that evaluated the annual economic burden attributable to RSV among US adults, burden was highest among adults with preexisting cardiac conditions. Conversely, immunocompromised individuals and those with chronic lung disease had lower odds of a concurrent acute cardiovascular event across RSV, influenza, and COVID-19 hospitalizations. This finding may reflect earlier presentation to care and prioritization for treatment (eg, increased antiviral uptake),^35,36^ which may potentially attenuate initial disease severity^37^ and thus subsequent risk of cardiovascular events, given greater awareness in these at-risk groups. Increasing awareness and vaccination uptake among adults with preexisting cardiac conditions is crucial, given effectiveness of existing influenza and COVID-19 vaccines in preventing RVI-related hospitalization and mitigating severe disease among adults with cardiac conditions,^38,39^ high frequency of congestive heart failure among RSV and influenza hospitalizations,^40^ and data affirming that simultaneous administration of RSV and influenza vaccination is safe in high-risk patients with heart failure, with a low incidence of mild adverse events.^41^ Given recent availability of RSV vaccination, older adults with preexisting cardiac history should be prioritized for vaccination against vaccine-preventable RVIs. Patients with a prior cardiac history might also benefit from more intensive clinical evaluation and monitoring for cardiac events during RSV hospitalization in light of greater risk.

### Strengths and Limitations

The strengths of our study include use of a comprehensive electronic health record–based health care claims database with national-level coverage to examine cardiovascular events in acute RSV, influenza, and COVID-19 hospitalizations, minimizing bias caused by loss to follow-up. Prevalence of acute cardiovascular events in RSV hospitalizations was compared against a contemporaneous control population (individuals hospitalized for influenza or COVID-19), allowing better contextualization of cardiovascular risk estimates for RSV hospitalizations vs other vaccine-preventable RVIs.

This study also has limitations. Reliance on administrative claims for ascertainment of cardiovascular events might have resulted in underreporting of milder events not affecting reimbursement, although most cardiovascular events that were evaluated in our study were serious enough to necessitate hospitalization. Mandatory reporting of COVID-19 hospitalizations may have biased toward inclusion of milder COVID-19 cases tested incidentally for SARS-CoV-2, whereas RSV and influenza hospitalizations were identified based on diagnosis codes in health care claims data and laboratory data were unavailable. However, our findings demonstrating higher odds of cardiovascular events in RSV vs COVID-19 hospitalizations remained robust when restricted to the subset of PCR-confirmed COVID-19 hospitalizations and when contemporaneous RSV and COVID-19 hospitalizations were compared. The effect of therapeutics on observed severity was not accounted for, although there is no routine treatment for RSV in adults and uptake of COVID-19 therapeutics was low (<5%).^36^ Additionally, our results may not be generalizable to other populations, particularly those with lower uptake of COVID-19 vaccination.

## Conclusions

In this cross-sectional study prior to RSV vaccination rollout, 1 in 10 RSV hospitalizations had a concurrent acute cardiovascular event, and odds of cardiovascular events were significantly higher in RSV hospitalizations vs COVID-19 hospitalizations in vaccine-boosted and unboosted individuals. There was no significant difference in acute cardiac events between RSV and influenza hospitalizations, except in contemporaneous RSV and influenza hospitalizations after the pandemic (2023-2024), in which odds of heart failure were significantly higher in RSV hospitalizations vs vaccine-breakthrough influenza. Evaluating vaccination’s role in attenuating risk of cardiovascular events associated with vaccine-preventable RVIs remains important given the availability of RSV vaccines for older adults and existing vaccine skepticism during COVID-19 endemicity. Individuals with a preexisting cardiac history remain at higher risk of acute cardiac events during RSV hospitalization and should be prioritized for vaccination.

## Notes

Supplement 1. **eTable 1.** Factors associated with occurrence of acute cardiovascular events amongst hospitalisations for respiratory-syncytial-virus in Singaporean adults **eTable 2.** Factors associated with occurrence of acute cardiovascular events amongst hospitalisations for influenza in Singaporean adults **eTable 3.** Factors associated with occurrence of acute cardiovascular events amongst hospitalisations for Omicron XBB/JN.1 SARS-CoV-2 in Singaporean adults **eTable 4.** Sociodemographic and clinical characteristics of hospitalisations for respiratory-syncytial-virus, influenza and Omicron XBB/JN.1 SARS-CoV-2 in Singaporean adults aged≥60 years **eTable 5.** Prevalence of acute cardiovascular events (composite) amongst hospitalisations for respiratory-syncytial-virus, influenza and Omicron XBB/JN.1 SARS-CoV-2 in Singaporean adults aged≥60 years **eTable 6.** Prevalence of acute cardiovascular events (individual) amongst hospitalisations for respiratory-syncytial-virus, influenza and Omicron XBB/JN.1 SARS-CoV-2 in Singaporean adults aged≥60 years **eTable 7.** Odds of intensive-care-unit (ICU) admission, stratified by presence of an acute cardiovascular event, during acute hospitalisations for respiratory-syncytial-virus, influenza and Omicron XBB/JN.1 SARS-CoV-2 in Singaporean adults aged≥60 years **eTable 8.** Sociodemographic and clinical characteristics of contemporaneous hospitalisations during 2023-2024 for respiratory-syncytial-virus, influenza and Omicron XBB/JN.1 SARS-CoV-2 in Singaporean adults **eTable 9.** Prevalence of acute cardiovascular events (composite) amongst contemporaneous hospitalisations during 2023-2024 for respiratory-syncytial-virus, influenza and Omicron XBB/JN.1 SARS-CoV-2 in Singaporean adults **eTable 10.** Prevalence of acute cardiovascular events (individual) amongst contemporaneous hospitalisations during 2023-2024 for respiratory-syncytial-virus, influenza and Omicron XBB/JN.1 SARS-CoV-2 in Singaporean adults **eTable 11.** Odds of intensive-care-unit (ICU) admission, stratified by presence of an acute cardiovascular event, amongst contemporaneous acute hospitalisations during 2023-2024 for respiratory-syncytial-virus, influenza and Omicron XBB/JN.1 SARS-CoV-2 in Singaporean adults **eTable 12.** Prevalence of acute cardiovascular events (composite) amongst hospitalisations for respiratory-syncytial-virus versus PCR- confirmed Omicron XBB/JN.1 SARS-CoV-2 in Singaporean adults **eTable 13.** Prevalence of acute cardiovascular events (individual) amongst hospitalisations for respiratory-syncytial-virus versus PCR-confirmed Omicron XBB/JN.1 SARS-CoV-2 in Singaporean adults **eAppendix.** List of ICD-10 codes used for outcomes of interest

Supplement 2.
Data Sharing Statement
