# Supplementary material for: Cardiac Events in Adults Hospitalized for Respiratory Syncytial Virus vs COVID-19 or Influenza
Source: JAMA Netw Open. 2025 May 22;8(5):e2511764. doi: 10.1001/jamanetworkopen.2025.11764 (PMC12100453; doi:10.1001/jamanetworkopen.2025.11764)
Supplement: Supplement 1. — eTable 1. Factors associated with occurrence of acute cardiovascular events amongst hospitalisations for respiratory-syncytial-virus in Singaporean adults eTable 2. Factors associated with occurrence of acute cardiovascular events amongst hospitalisations for influenza in Singaporean adults eTable 3. Factors associated with occurrence of acute cardiovascular events amongst hospitalisations for Omicron XBB/JN.1 SARS-CoV-2 in Singaporean adults eTable 4. Sociodemographic and clinical characteristics of hospitalisations for respiratory-syncytial-virus, influenza and Omicron XBB/JN.1 SARS-CoV-2 in Singaporean adults aged≥60 years eTable 5. Prevalence of acute cardiovascular events (composite) amongst hospitalisations for respiratory-syncytial-virus, influenza and Omicron XBB/JN.1 SARS-CoV-2 in Singaporean adults aged≥60 years eTable 6. Prevalence of acute cardiovascular events (individual) amongst hospitalisations for respiratory-syncytial-virus, influenza and Omicron XBB/JN.1 SARS-CoV-2 in Singaporean adults aged≥60 years eTable 7. Odds of intensive-care-unit (ICU) admission, stratified by presence of an acute cardiovascular event, during acute hospitalisations for respiratory-syncytial-virus, influenza and Omicron XBB/JN.1 SARS-CoV-2 in Singaporean adults aged≥60 years eTable 8. Sociodemographic and clinical characteristics of contemporaneous hospitalisations during 2023-2024 for respiratory-syncytial-virus, influenza and Omicron XBB/JN.1 SARS-CoV-2 in Singaporean adults eTable 9. Prevalence of acute cardiovascular events (composite) amongst contemporaneous hospitalisations during 2023-2024 for respiratory-syncytial-virus, influenza and Omicron XBB/JN.1 SARS-CoV-2 in Singaporean adults eTable 10. Prevalence of acute cardiovascular events (individual) amongst contemporaneous hospitalisations during 2023-2024 for respiratory-syncytial-virus, influenza and Omicron XBB/JN.1 SARS-CoV-2 in Singaporean adults eTable 11. Odds of intensive-care-unit (ICU) admission, stratified [file jamanetwopen-e2511764-s001.pdf]

Supplemental Online Content

Wee LE, Lim JT, Ho RWL, et al. Cardiac events in adults hospitalized for respiratory syncytial virus vs COVID-19 or influenza. *JAMA Netw Open*. 2025;8(5):e2511764. doi:10.1001/jamanetworkopen.2025.11764

eTable 1. Factors associated with occurrence of acute cardiovascular events amongst hospitalisations for respiratory-syncytial-virus in Singaporean adults
eTable 2. Factors associated with occurrence of acute cardiovascular events amongst hospitalisations for influenza in Singaporean adults
eTable 3. Factors associated with occurrence of acute cardiovascular events amongst hospitalisations for Omicron XBB/JN.1 SARS-CoV-2 in Singaporean adults
eTable 4. Sociodemographic and clinical characteristics of hospitalisations for respiratory-syncytial-virus, influenza and Omicron XBB/JN.1 SARS-CoV-2 in Singaporean adults aged≥60 years
eTable 5. Prevalence of acute cardiovascular events (composite) amongst hospitalisations for respiratory-syncytial-virus, influenza and Omicron XBB/JN.1 SARS-CoV-2 in Singaporean adults aged≥60 years
eTable 6. Prevalence of acute cardiovascular events (individual) amongst hospitalisations for respiratory-syncytial-virus, influenza and Omicron XBB/JN.1 SARS-CoV-2 in Singaporean adults aged≥60 years
eTable 7. Odds of intensive-care-unit (ICU) admission, stratified by presence of an acute cardiovascular event, during acute hospitalisations for respiratory-syncytial-virus, influenza and Omicron XBB/JN.1 SARS-CoV-2 in Singaporean adults aged≥60 years
eTable 8. Sociodemographic and clinical characteristics of contemporaneous hospitalisations during 2023-2024 for respiratory-syncytial-virus, influenza and Omicron XBB/JN.1 SARS-CoV-2 in Singaporean adults
eTable 9. Prevalence of acute cardiovascular events (composite) amongst contemporaneous hospitalisations during 2023-2024 for respiratory-syncytial-virus, influenza and Omicron XBB/JN.1 SARS-CoV-2 in Singaporean adults
eTable 10. Prevalence of acute cardiovascular events (individual) amongst contemporaneous hospitalisations during 2023-2024 for respiratory-syncytial-virus, influenza and Omicron XBB/JN.1 SARS-CoV-2 in Singaporean adults
eTable 11. Odds of intensive-care-unit (ICU) admission, stratified by presence of an acute cardiovascular event, amongst contemporaneous acute hospitalisations during 2023-2024 for respiratory-syncytial-virus, influenza and Omicron XBB/JN.1 SARS-CoV-2 in Singaporean adults
eTable 12. Prevalence of acute cardiovascular events (composite) amongst hospitalisations for respiratory-syncytial-virus versus PCR-confirmed Omicron XBB/JN.1 SARS-CoV-2 in Singaporean adults
eTable 13. Prevalence of acute cardiovascular events (individual) amongst hospitalisations for respiratory-syncytial-virus versus PCR-confirmed Omicron XBB/JN.1 SARS-CoV-2 in Singaporean adults
eAppendix. List of ICD-10 codes used for outcomes of interest

This supplemental material has been provided by the authors to give readers additional information about their work

© 2025 Wee LE et al. *JAMA Network Open.*

eTable 1: Factors associated with occurrence of acute cardiovascular events amongst hospitalisations for respiratory-syncytial-virus in Singaporean adults

Sociodemographic and clinical characteristics	Any cardiovascular event[a]		Any cardiac event[b]		Any major-cardiovascular-event, MACE[c]		Any heart failure		Any ischemic heart disease	
	N(%)	Adjusted odds ratio, aOR (95%CI)[d]	N(%)	Adjusted odds ratio, aOR (95%CI)[d]	N(%)	Adjusted odds ratio, aOR (95%CI)[d]	N(%)	Adjusted odds ratio, aOR (95%CI)[d]	N(%)	Adjusted odds ratio, aOR (95%CI)[d]
**Age, years**										
<60 years	30 (6.6%)	1.00 (.-.)	26 (5.7%)	1.00 (.-.)	15 (3.3%)	1.00 (.-.)	11 (2.4%)	1.00 (.-.)	6 (1.3%)	1.00 (.-.)
60-69 years	51 (12.1%)	1.64 (1.00-2.69)	48 (11.4%)	**1.83 (1.09-3.09)**	27 (6.4%)	1.56 (0.79-3.08)	15 (3.6%)	1.16 (0.50-2.68)	18 (4.3%)	2.57 (0.98-6.75)
70-79 years	60 (11.4%)	1.38 (0.85-2.26)	56 (10.6%)	1.52 (0.91-2.55)	26 (4.9%)	1.07 (0.54-2.14)	14 (2.7%)	0.72 (0.30-1.71)	16 (3.0%)	1.73 (0.64-4.68)
≥80 years	93 (12.5%)	1.41 (0.88-2.27)	90 (12.1%)	1.58 (0.96-2.61)	45 (6.0%)	1.19 (0.62-2.30)	26 (3.5%)	0.87 (0.39-1.96)	21 (2.8%)	1.57 (0.59-4.21)
**Ethnicity**										
Chinese	158 (11.9%)	1.00 (.-.)	148 (11.2%)	1.00 (.-.)	75 (5.7%)	1.00 (.-.)	43 (3.3%)	1.00 (.-.)	38 (2.9%)	1.00 (.-.)
Malay	55 (11.2%)	0.85 (0.60-1.21)	51 (10.3%)	0.84 (0.59-1.21)	28 (5.7%)	0.93 (0.58-1.50)	17 (3.4%)	1.02 (0.55-1.88)	16 (3.2%)	0.90 (0.48-1.71)
Others[e]	21 (6.3%)	**0.48 (0.29-0.79)**	21 (6.3%)	**0.51 (0.31-0.85)**	10 (3.0%)	0.52 (0.26-1.05)	6 (1.8%)	0.57 (0.23-1.38)	7 (2.1%)	0.57 (0.24-1.35)
**Gender**										
Female	127 (9.9%)	1.00 (.-.)	122 (9.5%)	1.00 (.-.)	62 (4.8%)	1.00 (.-.)	35 (2.7%)	1.00 (.-.)	34 (2.7%)	1.00 (.-.)
Male	107 (12.3%)	1.23 (0.92-1.63)	98 (11.3%)	1.16 (0.86-1.56)	51 (5.9%)	1.18 (0.79-1.75)	31 (3.6%)	1.26 (0.75-2.10)	27 (3.1%)	1.10 (0.65-1.87)
**Socioeconomic status (housing type)[f]**										
Public, 1-2 room	33 (12.9%)	1.00 (.-.)	31 (12.1%)	1.00 (.-.)	12 (4.7%)	1.00 (.-.)	6 (2.3%)	1.00 (.-.)	11 (4.3%)	1.00 (.-.)
Public, 3-room	53 (11.4%)	0.83 (0.52-1.35)	51 (11.0%)	0.87 (0.53-1.42)	25 (5.4%)	1.14 (0.55-2.35)	16 (3.4%)	1.49 (0.56-3.96)	18 (3.9%)	0.92 (0.42-2.04)
Public, 4-room	82 (12.3%)	0.95 (0.61-1.49)	78 (11.7%)	0.98 (0.62-1.56)	40 (6.0%)	1.33 (0.67-2.62)	20 (3.0%)	1.35 (0.53-3.46)	21 (3.1%)	0.75 (0.35-1.61)
Public, 5-room	54 (9.1%)	0.71 (0.44-1.15)	50 (8.4%)	0.72 (0.44-1.18)	28 (4.7%)	1.09 (0.53-2.22)	19 (3.2%)	1.56 (0.60-4.06)	9 (1.5%)	**0.37 (0.15-0.92)**
Private housing	12 (7.3%)	0.49 (0.24-1.01)	10 (6.1%)	**0.44 (0.20-0.95)**	8 (4.9%)	1.02 (0.39-2.63)	5 (3.0%)	1.31 (0.38-4.58)	2 (1.2%)	0.28 (0.06-1.34)
**Comorbidity burden[g]**										
Charlson Comorbidity Index, CCMI=0	43 (8.2%)	1.00 (.-.)	42 (8.0%)	1.00 (.-.)	17 (3.2%)	1.00 (.-.)	13 (2.5%)	1.00 (.-.)	7 (1.3%)	1.00 (.-.)
Mild (CCMI=1-2)	123 (10.4%)	1.21 (0.78-1.89)	112 (9.4%)	1.08 (0.69-1.71)	61 (5.1%)	1.68 (0.87-3.24)	30 (2.5%)	0.98 (0.44-2.21)	36 (3.0%)	1.76 (0.67-4.60)
Moderate-severe (CCMI≥3)	68 (15.6%)	1.50 (0.82-2.76)	66 (15.1%)	1.40 (0.75-2.62)	35 (8.0%)	2.21 (0.93-5.25)	23 (5.3%)	1.58 (0.55-4.57)	18 (4.1%)	1.63 (0.47-5.60)
**Immunocompromised[h]**										
No	187 (11.1%)	1.00 (.-.)	180 (10.7%)	1.00 (.-.)	92 (5.5%)	1.00 (.-.)	53 (3.2%)	1.00 (.-.)	49 (2.9%)	1.00 (.-.)
Yes	47 (10.1%)	0.74 (0.50-1.07)	40 (8.6%)	**0.65 (0.44-0.97)**	21 (4.5%)	0.59 (0.35-1.00)	13 (2.8%)	0.68 (0.35-1.33)	12 (2.6%)	0.67 (0.33-1.35)
**Pre-existing cardiac history[i]**										
No	121 (7.7%)	1.00 (.-.)	109 (6.9%)	1.00 (.-.)	56 (3.5%)	1.00 (.-.)	27 (1.7%)	1.00 (.-.)	31 (2.0%)	1.00 (.-.)

© 2025 Wee LE et al. *JAMA Network Open.*

Yes	113 (19.9%)	**2.53 (1.84-3.48)**	111 (19.5%)	**2.76 (1.99-3.82)**	57 (10.0%)	**2.46 (1.59-3.83)**	39 (6.9%)	**4.05 (2.25-7.31)**	30 (5.3%)	**2.22 (1.23-3.99)**
**Pre-existing dyslipidemia**										
No	209 (10.4%)	1.00 (.-.)	195 (9.7%)	1.00 (.-.)	100 (5.0%)	1.00 (.-.)	61 (3.0%)	1.00 (.-.)	52 (2.6%)	1.00 (.-.)
Yes	25 (18.5%)	1.47 (0.90-2.42)	25 (18.5%)	1.57 (0.95-2.57)	13 (9.6%)	1.46 (0.76-2.81)	5 (3.7%)	0.75 (0.28-1.99)	9 (6.7%)	2.03 (0.92-4.45)
**Pre-existing diabetes**										
No	131 (9.9%)	1.00 (.-.)	123 (9.3%)	1.00 (.-.)	56 (4.3%)	1.00 (.-.)	34 (2.6%)	1.00 (.-.)	26 (2.0%)	1.00 (.-.)
Yes	103 (12.4%)	0.85 (0.60-1.21)	97 (11.7%)	0.84 (0.58-1.21)	57 (6.9%)	1.02 (0.63-1.65)	32 (3.9%)	1.02 (0.53-1.94)	35 (4.2%)	1.46 (0.76-2.78)
**Pre-existing chronic lung disease**										
No	192 (11.9%)	1.00 (.-.)	179 (11.0%)	1.00 (.-.)	99 (6.1%)	1.00 (.-.)	61 (3.8%)	1.00 (.-.)	49 (3.0%)	1.00 (.-.)
Yes	42 (8.0%)	**0.50 (0.34-0.74)**	41 (7.8%)	**0.52 (0.35-0.78)**	14 (2.7%)	**0.32 (0.17-0.58)**	5 (0.9%)	**0.20 (0.08-0.53)**	12 (2.3%)	0.58 (0.29-1.15)
**Year of infection**										
2017-2022	67 (8.6%)	1.00 (.-.)	62 (8.0%)	1.00 (.-.)	37 (4.8%)	1.00 (.-.)	19 (2.4%)	1.00 (.-.)	26 (3.3%)	1.00 (.-.)
2023-2024	167 (12.2%)	1.15 (0.84-1.58)	158 (11.5%)	1.15 (0.83-1.59)	76 (5.5%)	0.88 (0.58-1.35)	47 (3.4%)	1.14 (0.64-2.02)	35 (2.6%)	**0.54 (0.31-0.93)**

[a] Any cardiovascular event defined as the composite of any cardiac, cerebrovascular or thrombotic event
[b] Any cardiac event defined as the composite of any dysrhythmia, ischemic heart disease, heart failure, or other cardiac event
[c] Major-adverse-cardiovascular/cerebrovascular-event (MACE) was defined as the first incidence of myocardial infarction, stroke, heart failure, ventricular arrhythmia, or sudden cardiovascular collapse.
[d] Odds ratio estimated using multivariate logistic regression models, adjusting for age, gender, ethnicity, socioeconomic status, comorbidity burden (Charlson Comorbidity Index), immunocompromised status, pre-existing cardiac history, pre-existing diabetes, preexisting dyslipidemia, pre-existing chronic lung disease, year of infection.
[e] Including individuals of Indian ethnicity, mixed ethnicity or other ethnicities (eg. Eurasian, Arab); categories consolidated as individual numbers too small for separate analysis.
[f] Housing type was used as an indicator of socioeconomic status.
[g] Comorbidity burden was defined using the Charlson Comorbidity Index (CCMI), which consists of the following comorbidities: myocardial infarction, chronic heart failure, peripheral vascular disease, cerebrovascular accident, dementia, chronic obstructive pulmonary disease, connective tissue disease, peptic ulcer disease, diabetes mellitus, hemiplegia, liver disease, moderate to severe renal impairment, solid tumor, leukemia, human immunodeficiency virus (HIV) infection with AIDS.
[h] Immunocompromised status was defined as: presence of solid malignancy, hematologic malignancy, rheumatologic or inflammatory disorders, organ or stem cell transplant, or other intrinsic immune condition or immunodeficiency.
[i] Pre-existing cardiac history was defined as history of ischemic heart disease or heart failure.

© 2025 Wee LE et al. *JAMA Network Open.*

eTable 2: Factors associated with occurrence of acute cardiovascular events amongst hospitalisations for influenza in Singaporean adults

Sociodemographic and clinical characteristics	Any cardiovascular event[a]		Any cardiac event[b]		Any major-cardiovascular-event, MACE[c]		Any heart failure		Any ischemic heart disease	
	**N(%)**	**Adjusted odds ratio, aOR (95%CI)[d]**	**N(%)**	**Adjusted odds ratio, aOR (95%CI)[d]**	**N(%)**	**Adjusted odds ratio, aOR (95%CI)[d]**	**N(%)**	**Adjusted odds ratio, aOR (95%CI)[d]**	**N(%)**	**Adjusted odds ratio, aOR (95%CI)[d]**
**Age, years**										
<60 years	255 (4.2%)	1.00 (.-.)	231 (3.8%)	1.00 (.-.)	86 (1.4%)	1.00 (.-.)	37 (0.6%)	1.00 (.-.)	50 (0.8%)	1.00 (.-.)
60-69 years	265 (9.7%)	**2.06 (1.70-2.49)**	240 (8.8%)	**2.02 (1.66-2.47)**	122 (4.4%)	**2.23 (1.66-3.00)**	54 (2.0%)	**1.88 (1.21-2.94)**	95 (3.5%)	**3.29 (2.29-4.72)**
70-79 years	302 (9.9%)	**2.10 (1.74-2.54)**	268 (8.8%)	**2.03 (1.67-2.48)**	143 (4.7%)	**2.39 (1.79-3.21)**	50 (1.6%)	1.56 (0.99-2.48)	100 (3.3%)	**3.28 (2.28-4.74)**
≥80 years	318 (12.8%)	**2.74 (2.26-3.32)**	288 (11.6%)	**2.72 (2.22-3.33)**	171 (6.9%)	**3.51 (2.62-4.69)**	84 (3.4%)	**2.99 (1.93-4.61)**	80 (3.2%)	**3.34 (2.27-4.92)**
**Ethnicity**										
Chinese	667 (7.9%)	1.00 (.-.)	595 (7.0%)	1.00 (.-.)	293 (3.5%)	1.00 (.-.)	133 (1.6%)	1.00 (.-.)	168 (2.0%)	1.00 (.-.)
Malay	315 (8.9%)	**1.23 (1.06-1.43)**	288 (8.2%)	**1.25 (1.07-1.46)**	155 (4.4%)	**1.42 (1.15-1.76)**	65 (1.8%)	1.16 (0.84-1.61)	105 (3.0%)	**1.63 (1.25-2.12)**
Others[e]	158 (6.7%)	0.93 (0.77-1.12)	144 (6.1%)	0.94 (0.77-1.14)	74 (3.1%)	1.02 (0.77-1.33)	27 (1.1%)	0.72 (0.47-1.11)	52 (2.2%)	1.25 (0.90-1.73)
**Gender**										
Female	521 (6.7%)	1.00 (.-.)	466 (6.0%)	1.00 (.-.)	226 (2.9%)	1.00 (.-.)	108 (1.4%)	1.00 (.-.)	114 (1.5%)	1.00 (.-.)
Male	619 (9.3%)	**1.28 (1.13-1.45)**	561 (8.4%)	**1.29 (1.13-1.47)**	296 (4.4%)	**1.38 (1.15-1.66)**	117 (1.8%)	1.06 (0.81-1.40)	211 (3.2%)	**1.96 (1.55-2.48)**
**Socioeconomic status (housing type)[f]**										
Public, 1-2 room	148 (9.2%)	1.00 (.-.)	133 (8.2%)	1.00 (.-.)	77 (4.8%)	1.00 (.-.)	34 (2.1%)	1.00 (.-.)	45 (2.8%)	1.00 (.-.)
Public, 3-room	273 (9.6%)	1.10 (0.88-1.36)	251 (8.8%)	1.13 (0.90-1.42)	129 (4.5%)	1.00 (0.74-1.35)	56 (2.0%)	0.95 (0.61-1.48)	91 (3.2%)	1.34 (0.92-1.94)
Public, 4-room	370 (8.2%)	1.03 (0.84-1.27)	334 (7.4%)	1.05 (0.84-1.30)	155 (3.4%)	0.87 (0.65-1.15)	72 (1.6%)	0.90 (0.59-1.38)	97 (2.1%)	1.00 (0.69-1.44)
Public, 5-room	274 (6.3%)	0.87 (0.70-1.08)	245 (5.6%)	0.87 (0.70-1.10)	114 (2.6%)	0.78 (0.57-1.06)	45 (1.0%)	0.72 (0.45-1.15)	71 (1.6%)	0.89 (0.60-1.31)
Private housing	75 (7.3%)	0.91 (0.67-1.22)	64 (6.2%)	0.86 (0.62-1.18)	47 (4.6%)	1.24 (0.84-1.84)	18 (1.8%)	1.07 (0.58-1.95)	21 (2.0%)	0.96 (0.56-1.64)
**Comorbidity burden[g]**										
Charlson Comorbidity Index, CCMI=0	396 (5.7%)	1.00 (.-.)	359 (5.2%)	1.00 (.-.)	127 (1.8%)	1.00 (.-.)	43 (0.6%)	1.00 (.-.)	93 (1.3%)	1.00 (.-.)
Mild (CCMI=1-2)	544 (9.0%)	1.12 (0.93-1.36)	481 (8.0%)	1.01 (0.82-1.24)	262 (4.3%)	**1.57 (1.18-2.09)**	112 (1.9%)	**2.05 (1.31-3.22)**	155 (2.6%)	1.01 (0.71-1.44)
Moderate-severe (CCMI≥3)	200 (14.1%)	1.28 (0.95-1.73)	187 (13.1%)	1.13 (0.82-1.54)	133 (9.3%)	**2.46 (1.65-3.69)**	70 (4.9%)	**3.58 (1.97-6.52)**	77 (5.4%)	1.33 (0.80-2.22)
**Immunocompromised[h]**										
No	997 (8.0%)	1.00 (.-.)	897 (7.2%)	1.00 (.-.)	436 (3.5%)	1.00 (.-.)	192 (1.5%)	1.00 (.-.)	269 (2.2%)	1.00 (.-.)
Yes	143 (7.5%)	**0.68 (0.56-0.84)**	130 (6.8%)	**0.71 (0.58-0.88)**	86 (4.5%)	0.82 (0.63-1.06)	33 (1.7%)	**0.62 (0.41-0.92)**	56 (2.9%)	1.02 (0.74-1.41)
**Pre-existing cardiac history[i]**										
No	762 (6.1%)	1.00 (.-.)	662 (5.3%)	1.00 (.-.)	321 (2.6%)	1.00 (.-.)	106 (0.9%)	1.00 (.-.)	199 (1.6%)	1.00 (.-.)

© 2025 Wee LE et al. *JAMA Network Open.*

Yes	378 (19.3%)	**2.86 (2.44-3.34)**	365 (18.6%)	**3.27 (2.78-3.85)**	201 (10.2%)	**2.62 (2.11-3.24)**	119 (6.1%)	**4.67 (3.41-6.38)**	126 (6.4%)	**2.79 (2.13-3.66)**
**Pre-existing dyslipidemia**										
No	1074 (7.8%)	1.00 (.-.)	962 (6.9%)	1.00 (.-.)	486 (3.5%)	1.00 (.-.)	204 (1.5%)	1.00 (.-.)	298 (2.2%)	1.00 (.-.)
Yes	66 (12.1%)	0.93 (0.70-1.23)	65 (11.9%)	0.99 (0.75-1.32)	36 (6.6%)	0.97 (0.67-1.40)	21 (3.8%)	1.18 (0.73-1.92)	27 (4.9%)	1.19 (0.77-1.82)
**Pre-existing diabetes**										
No	710 (6.9%)	1.00 (.-.)	636 (6.2%)	1.00 (.-.)	272 (2.7%)	1.00 (.-.)	110 (1.1%)	1.00 (.-.)	179 (1.8%)	1.00 (.-.)
Yes	430 (10.3%)	0.91 (0.76-1.08)	391 (9.4%)	0.96 (0.80-1.15)	250 (6.0%)	1.05 (0.82-1.33)	115 (2.8%)	0.98 (0.69-1.39)	146 (3.5%)	1.07 (0.79-1.45)
**Pre-existing chronic lung disease**										
No	968 (7.8%)	1.00 (.-.)	868 (7.0%)	1.00 (.-.)	446 (3.6%)	1.00 (.-.)	195 (1.6%)	1.00 (.-.)	268 (2.2%)	1.00 (.-.)
Yes	172 (8.5%)	**0.78 (0.64-0.94)**	159 (7.9%)	0.82 (0.67-1.01)	76 (3.8%)	**0.62 (0.47-0.82)**	30 (1.5%)	**0.51 (0.33-0.78)**	57 (2.8%)	0.85 (0.61-1.19)
**Year of infection**										
2017-2022	528 (6.9%)	1.00 (.-.)	481 (6.3%)	1.00 (.-.)	244 (3.2%)	1.00 (.-.)	111 (1.5%)	1.00 (.-.)	165 (2.2%)	1.00 (.-.)
2023-2024	612 (9.1%)	1.06 (0.93-1.20)	546 (8.1%)	1.01 (0.88-1.16)	278 (4.1%)	0.92 (0.77-1.11)	114 (1.7%)	**0.73 (0.55-0.97)**	160 (2.4%)	**0.77 (0.61-0.97)**
**Vaccinated for influenza[j]**										
Vaccinated	914 (7.6%)	1.00 (.-.)	821 (6.9%)	1.00 (.-.)	421 (3.5%)	1.00 (.-.)	185 (1.5%)	1.00 (.-.)	255 (2.1%)	1.00 (.-.)
Unvaccinated	226 (9.4%)	0.95 (0.81-1.12)	206 (8.5%)	0.96 (0.81-1.14)	101 (4.2%)	0.85 (0.67-1.07)	40 (1.7%)	0.76 (0.53-1.10)	70 (2.9%)	1.00 (0.75-1.33)

[a] Any cardiovascular event defined as the composite of any cardiac, cerebrovascular or thrombotic event
[b] Any cardiac event defined as the composite of any dysrhythmia, ischemic heart disease, heart failure, or other cardiac event
[c] Major-adverse-cardiovascular/cerebrovascular-event (MACE) was defined as the first incidence of myocardial infarction, stroke, heart failure, ventricular arrhythmia, or sudden cardiovascular collapse.
[d] Odds ratio estimated using multivariate logistic regression models, adjusting for age, gender, ethnicity, socioeconomic status, comorbidity burden (Charlson Comorbidity Index), immunocompromised status, pre-existing cardiac history, pre-existing diabetes, preexisting dyslipidemia, pre-existing chronic lung disease, year of infection.
[e] Including individuals of Indian ethnicity, mixed ethnicity or other ethnicities (eg. Eurasian, Arab); categories consolidated as individual numbers too small for separate analysis.
[f] Housing type was used as an indicator of socioeconomic status.
[g] Comorbidity burden was defined using the Charlson Comorbidity Index (CCMI), which consists of the following comorbidities: myocardial infarction, chronic heart failure, peripheral vascular disease, cerebrovascular accident, dementia, chronic obstructive pulmonary disease, connective tissue disease, peptic ulcer disease, diabetes mellitus, hemiplegia, liver disease, moderate to severe renal impairment, solid tumor, leukemia, human immunodeficiency virus (HIV) infection with AIDS.
[h] Immunocompromised status was defined as: presence of solid malignancy, hematologic malignancy, rheumatologic or inflammatory disorders, organ or stem cell transplant, or other intrinsic immune condition or immunodeficiency.
[i] Pre-existing cardiac history was defined as history of ischemic heart disease or heart failure.
[j] Individuals who had received seasonal influenza vaccination <365 days prior to hospitalisation were considered vaccinated.

© 2025 Wee LE et al. *JAMA Network Open.*

eTable 3: Factors associated with occurrence of acute cardiovascular events amongst hospitalisations for Omicron XBB/JN.1 SARS-CoV-2 in Singaporean adults

**Sociodemographic and clinical characteristics**	**Any cardiovascular event[a]**		**Any cardiac event[b]**		**Any major-cardiovascular-event, MACE[c]**		**Any heart failure**		**Any ischemic heart disease**	
	**N(%)**	**Adjusted odds ratio, aOR (95%CI)[d]**	**N(%)**	**Adjusted odds ratio, aOR (95%CI)[d]**	**N(%)**	**Adjusted odds ratio, aOR (95%CI)[d]**	**N(%)**	**Adjusted odds ratio, aOR (95%CI)[d]**	**N(%)**	**Adjusted odds ratio, aOR (95%CI)[d]**
**Age, years**										
<60 years	182 (6.2%)	1.00 (.-.)	147 (5.0%)	1.00 (.-.)	93 (3.2%)	1.00 (.-.)	37 (1.3%)	1.00 (.-.)	64 (2.2%)	1.00 (.-.)
60-69 years	263 (9.2%)	**1.28 (1.04-1.57)**	194 (6.8%)	1.10 (0.88-1.39)	159 (5.6%)	**1.41 (1.07-1.84)**	52 (1.8%)	1.01 (0.65-1.57)	91 (3.2%)	1.10 (0.79-1.55)
70-79 years	387 (8.5%)	1.20 (0.99-1.46)	312 (6.9%)	1.17 (0.94-1.44)	223 (4.9%)	1.24 (0.96-1.61)	68 (1.5%)	0.91 (0.59-1.38)	136 (3.0%)	1.06 (0.77-1.45)
≥80 years	506 (8.3%)	1.16 (0.96-1.41)	419 (6.9%)	1.15 (0.93-1.41)	298 (4.9%)	1.27 (0.99-1.64)	96 (1.6%)	0.95 (0.63-1.42)	162 (2.7%)	0.97 (0.71-1.33)
**Ethnicity**										
Chinese	980 (7.9%)	1.00 (Ref)	766 (6.1%)	1.00 (Ref)	562 (4.5%)	1.00 (Ref)	161 (1.3%)	1.00 (Ref)	321 (2.6%)	1.00 (Ref)
Malay	237 (10.1%)	**1.27 (1.09-1.49)**	198 (8.5%)	**1.33 (1.12-1.58)**	140 (6.0%)	**1.25 (1.02-1.53)**	62 (2.6%)	**1.70 (1.23-2.33)**	80 (3.4%)	1.20 (0.93-1.57)
Others[e]	121 (7.5%)	0.90 (0.73-1.10)	108 (6.7%)	1.01 (0.81-1.25)	71 (4.4%)	0.91 (0.70-1.18)	30 (1.9%)	1.20 (0.80-1.80)	52 (3.2%)	1.13 (0.83-1.54)
**Gender**										
Female	591 (7.3%)	1.00 (Ref)	470 (5.8%)	1.00 (Ref)	329 (4.1%)	1.00 (Ref)	111 (1.4%)	1.00 (Ref)	175 (2.2%)	1.00 (Ref)
Male	747 (8.9%)	1.11 (0.99-1.25)	602 (7.2%)	1.11 (0.98-1.27)	444 (5.3%)	**1.17 (1.01-1.36)**	142 (1.7%)	1.01 (0.79-1.31)	278 (3.3%)	**1.38 (1.14-1.68)**
**Socioeconomic status (housing type)[f]**										
Public, 1-2 room	154 (8.3%)	1.00 (Ref)	134 (7.2%)	1.00 (Ref)	88 (4.7%)	1.00 (Ref)	42 (2.3%)	1.00 (Ref)	44 (2.4%)	1.00 (Ref)
Public, 3-room	286 (7.8%)	1.02 (0.83-1.26)	225 (6.1%)	0.94 (0.75-1.18)	178 (4.8%)	1.13 (0.87-1.48)	48 (1.3%)	0.69 (0.45-1.06)	106 (2.9%)	1.40 (0.97-2.01)
Public, 4-room	436 (8.6%)	1.19 (0.97-1.44)	354 (6.9%)	1.14 (0.92-1.41)	244 (4.8%)	1.16 (0.90-1.49)	85 (1.7%)	0.93 (0.64-1.37)	154 (3.0%)	**1.52 (1.07-2.15)**
Public, 5-room	373 (8.4%)	1.20 (0.98-1.47)	283 (6.3%)	1.08 (0.86-1.35)	224 (5.0%)	1.29 (0.99-1.67)	71 (1.6%)	0.98 (0.65-1.46)	116 (2.6%)	1.37 (0.96-1.97)
Private housing	89 (6.7%)	0.98 (0.74-1.30)	76 (5.8%)	1.01 (0.75-1.37)	39 (3.0%)	0.77 (0.52-1.14)	7 (0.5%)	0.35 (0.16-0.80)	33 (2.5%)	1.44 (0.90-2.31)
**Comorbidity burden[g]**										
Charlson Comorbidity Index, CCMI=0	261 (5.8%)	1.00 (Ref)	193 (4.3%)	1.00 (Ref)	124 (2.8%)	1.00 (Ref)	28 (0.6%)	1.00 (Ref)	73 (1.6%)	1.00 (Ref)
Mild (CCMI=1-2)	663 (7.9%)	1.10 (0.92-1.32)	531 (6.3%)	1.13 (0.93-1.39)	383 (4.6%)	**1.30 (1.02-1.66)**	120 (1.4%)	**2.03 (1.28-3.23)**	221 (2.6%)	1.03 (0.75-1.43)
Moderate-severe (CCMI≥3)	414 (11.7%)	1.20 (0.95-1.52)	348 (9.8%)	1.20 (0.92-1.56)	266 (7.5%)	**1.60 (1.17-2.18)**	105 (3.0%)	**3.02 (1.74-5.26)**	159 (4.5%)	1.06 (0.71-1.59)
**Immunocompromised[h]**										
No	1076 (8.1%)	1.00 (Ref)	879 (6.6%)	1.00 (Ref)	628 (4.7%)	1.00 (Ref)	214 (1.6%)	1.00 (Ref)	378 (2.8%)	1.00 (Ref)
Yes	262 (8.3%)	0.91 (0.78-1.06)	193 (6.1%)	**0.81 (0.68-0.96)**	145 (4.6%)	**0.81 (0.66-0.99)**	39 (1.2%)	**0.58 (0.40-0.83)**	75 (2.4%)	0**.73 (0.56-0.96)**
**Pre-existing cardiac history[i]**										

© 2025 Wee LE et al. *JAMA Network Open.*

No	708 (5.7%)	1.00 (Ref)	510 (4.1%)	1.00 (Ref)	419 (3.3%)	1.00 (Ref)	95 (0.8%)	1.00 (Ref)	209 (1.7%)	1.00 (Ref)
Yes	630 (16.2%)	**2.96 (2.61-3.37)**	562 (14.4%)	**3.65 (3.18-4.21)**	354 (9.1%)	**2.37 (2.01-2.78)**	158 (4.1%)	**4.23 (3.18-5.63)**	244 (6.3%)	**3.49 (2.83-4.31)**
**Pre-existing dyslipidemia**										
No	1201 (7.8%)	1.00 (Ref)	958 (6.2%)	1.00 (Ref)	688 (4.5%)	1.00 (Ref)	222 (1.4%)	1.00 (Ref)	399 (2.6%)	1.00 (Ref)
Yes	137 (13.5%)	**1.27 (1.04-1.55)**	114 (11.2%)	**1.25 (1.01-1.56)**	85 (8.4%)	**1.32 (1.04-1.69)**	31 (3.1%)	1.22 (0.82-1.81)	54 (5.3%)	1.28 (0.95-1.74)
**Pre-existing diabetes**										
No	701 (7.1%)	1.00 (Ref)	551 (5.6%)	1.00 (Ref)	357 (3.6%)	1.00 (Ref)	121 (1.2%)	1.00 (Ref)	189 (1.9%)	1.00 (Ref)
Yes	637 (9.7%)	1.03 (0.89-1.19)	521 (7.9%)	1.03 (0.88-1.21)	416 (6.3%)	**1.21 (1.01-1.46)**	132 (2.0%)	0.81 (0.60-1.09)	264 (4.0%)	**1.62 (1.27-2.06)**
**Pre-existing chronic lung disease**										
No	1188 (8.2%)	1.00 (Ref)	935 (6.5%)	1.00 (Ref)	687 (4.8%)	1.00 (Ref)	217 (1.5%)	1.00 (Ref)	407 (2.8%)	1.00 (Ref)
Yes	150 (7.6%)	**0.73 (0.61-0.88)**	137 (6.9%)	0.83 (0.68-1.01)	86 (4.3%)	**0.70 (0.55-0.89)**	36 (1.8%)	0.73 (0.50-1.06)	46 (2.3%)	**0.67 (0.48-0.93)**
**Vaccination status**										
Unboosted[j]	980 (8.2%)	1.00 (Ref)	789 (6.6%)	1.00 (Ref)	563 (4.7%)	1.00 (Ref)	188 (1.6%)	1.00 (Ref)	342 (2.9%)	1.00 (Ref)
Boosted ≥ 1 year prior with ancestral mRNA vaccines[j]	200 (7.8%)	1.03 (0.88-1.21)	152 (5.9%)	0.99 (0.82-1.19)	118 (4.6%)	1.09 (0.88-1.34)	35 (1.4%)	1.05 (0.73-1.52)	63 (2.5%)	0.95 (0.72-1.25)
Boosted <1 year prior with updated mRNA vaccines[j]	158 (8.2%)	0.93 (0.78-1.11)	131 (6.8%)	0.94 (0.77-1.14)	92 (4.7%)	0.96 (0.76-1.21)	30 (1.5%)	0.87 (0.59-1.29)	48 (2.5%)	0.81 (0.59-1.10)
**Required supplemental oxygen**										
No	1214 (7.9%)	1.00 (Ref)	963 (6.2%)	1.00 (Ref)	700 (4.5%)	1.00 (Ref)	230 (1.5%)	1.00 (Ref)	409 (2.7%)	1.00 (Ref)
Yes	124 (12.3%)	1.57 (1.28-1.93)	109 (10.8%)	**1.70 (1.37-2.11)**	73 (7.2%)	**1.56 (1.21-2.01)**	23 (2.3%)	1.37 (0.88-2.13)	44 (4.4%)	**1.57 (1.14-2.18)**

[a] Any cardiovascular event defined as the composite of any cardiac, cerebrovascular or thrombotic event

[b] Any cardiac event defined as the composite of any dysrhythmia, ischemic heart disease, heart failure, or other cardiac event

[c] Major-adverse-cardiovascular/cerebrovascular-event (MACE) was defined as the first incidence of myocardial infarction, stroke, heart failure, ventricular arrhythmia, or sudden cardiovascular collapse.

[d] Odds ratio estimated using multivariate logistic regression models, adjusting for age, gender, ethnicity, socioeconomic status, comorbidity burden (Charlson Comorbidity Index), immunocompromised status, pre-existing cardiac history, pre-existing diabetes, preexisting dyslipidemia, pre-existing chronic lung disease, year of infection.

[e] Including individuals of Indian ethnicity, mixed ethnicity or other ethnicities (eg. Eurasian, Arab); categories consolidated as individual numbers too small for separate analysis.

[f] Housing type was used as an indicator of socioeconomic status.

[g] Comorbidity burden was defined using the Charlson Comorbidity Index (CCMI), which consists of the following comorbidities: myocardial infarction, chronic heart failure, peripheral vascular disease, cerebrovascular accident, dementia, chronic obstructive pulmonary disease, connective tissue disease, peptic ulcer disease, diabetes mellitus, hemiplegia, liver disease, moderate to severe renal impairment, solid tumor, leukemia, human immunodeficiency virus (HIV) infection with AIDS.

[h] Immunocompromised status was defined as: presence of solid malignancy, hematologic malignancy, rheumatologic or inflammatory disorders, organ or stem cell transplant, or other intrinsic immune condition or immunodeficiency.

[i] Pre-existing cardiac history was defined as history of ischemic heart disease or heart failure.

[j] Boosting was defined as having received at least a third mRNA vaccine dose ≥6 months following completion of an initial two-dose mRNA primary vaccine series.

© 2025 Wee LE et al. *JAMA Network Open.*

eTable 4: Sociodemographic and clinical characteristics of hospitalisations for respiratory-syncytial-virus, influenza and Omicron XBB/JN.1 SARS-CoV-2 in Singaporean adults aged≥60 years

Sociodemographic and clinical characteristics	Respiratory-syncytial-virus (RSV) hospitalisations, 2017-2024[a] N(%)	Influenza hospitalisations, 2017-2024, vaccinated,[b] N(%)	Influenza hospitalisations, 2017-2024, unvaccinated,[b] N(%)	Omicron XBB/JN.1 COVID-19 hospitalisations, 2023-2024	
				**Boosted,[c] N(%)**	**Unboosted,[c] N(%)**
Total number of hospitalisations, N(%)	1,694 (7.2%)	1,885 (8.0%)	6,390 (27.3%)	11,890 (50.7%)	1,584 (6.8%)
**Age, years**					
Mean age, years (S.D)	77.5 (9.842)	75.7 (8.395)	74.2 (9.171)	77.8 (9.281)	79.6 (10.150)
60-69 years	421 (24.9%)	488 (25.9%)	2,254 (35.3%)	2,550 (21.4%)	301 (19.0%)
70-79 years	528 (31.2%)	782 (41.5%)	2,258 (35.3%)	4,083 (34.3%)	453 (28.6%)
≥80 years	745 (44.0%)	615 (32.6%)	1,878 (29.4%)	5,257 (44.2%)	830 (52.4%)
**Ethnicity**					
Chinese	1,106 (65.3%)	1,274 (67.6%)	4,050 (63.4%)	9,436 (79.4%)	1,211 (76.5%)
Malay	354 (20.9%)	376 (19.9%)	1,476 (23.1%)	1,511 (12.7%)	183 (11.6%)
Others[d]	234 (13.8%)	235 (12.5%)	864 (13.5%)	943 (7.9%)	190 (12.0%)
**Gender**					
Female	1,009 (59.6%)	872 (46.3%)	3,330 (52.1%)	5,644 (47.5%)	827 (52.2%)
**Socioeconomic status (housing type)[e]**					
Public, 1-2 room	197 (11.6%)	224 (11.9%)	870 (13.6%)	1,387 (11.7%)	200 (12.6%)
Public, 3-room	383 (22.6%)	420 (22.3%)	1,487 (23.3%)	2,762 (23.2%)	379 (23.9%)
Public, 4-room	520 (30.7%)	614 (32.6%)	1,954 (30.6%)	3,592 (30.2%)	503 (31.8%)
Public, 5-room	448 (26.4%)	475 (25.2%)	1,596 (25.0%)	3,117 (26.2%)	372 (23.5%)
Private housing	146 (8.6%)	152 (8.1%)	483 (7.6%)	1,032 (8.7%)	130 (8.2%)
**Comorbidity burden[f]**					
Charlson Comorbidity Index, CCMI=0	299 (17.7%)	343 (18.2%)	2,129 (33.3%)	2,560 (21.5%)	319 (20.1%)
Mild (CCMI=1-2)	990 (58.4%)	1,155 (61.3%)	3,410 (53.4%)	6,518 (54.8%)	812 (51.3%)
Moderate-severe (CCMI≥3)	405 (23.9%)	387 (20.5%)	851 (13.3%)	2,812 (23.7%)	453 (28.6%)
**Immunocompromised[g]**					
Yes	380 (22.4%)	398 (21.1%)	1,022 (16.0%)	2,320 (19.5%)	348 (22.0%)
**Pre-existing cardiac history[h]**					
Yes	520 (30.7%)	448 (23.8%)	1,150 (18.0%)	2,964 (24.9%)	497 (31.4%)
**Pre-existing dyslipidemia**					
Yes	112 (6.6%)	125 (6.6%)	287 (4.5%)	768 (6.5%)	98 (6.2%)
**Pre-existing diabetes**					
Yes	746 (44.0%)	868 (46.0%)	2,630 (41.2%)	5,340 (44.9%)	668 (42.2%)
**Pre-existing chronic lung disease**					
Yes	441 (26.0%)	578 (30.7%)	855 (13.4%)	1,515 (12.7%)	211 (13.3%)

© 2025 Wee LE et al. *JAMA Network Open.*

**Clinical outcomes and healthcare utilisation**					
Required intensive-care-unit (ICU) admission	70 (4.1%)	72 (3.8%)	269 (4.2%)	293 (2.5%)	46 (2.9%)
Length-of-stay, days (median, IQR)	5.0 (3.0-8.0)	4.0 (3.0-7.0)	4.0 (3.0-7.0)	4.0 (2.0-9.0)	5.0 (3.0-10.0)
ICU length-of-stay, days (median, IQR)	3.0 (2.0-5.0)	3.0 (1.0-5.5)	3.0 (1.0-5.0)	3.0 (1.0-5.0)	3.0 (1.0-10.0)

[a] Hospitalisations attributed to respiratory-syncytial-virus, RSV, from January 2017- June 2024.
[b] Hospitalisations attributed to influenza from January 2017- June 2024. Individuals who had received seasonal influenza vaccination <365 days prior to hospitalisation were considered vaccinated.
[c] Hospitalisations attributed to COVID-19 in January 2023-June 2024; during 2023-2024, community transmission of COVID-19 was dominated by various Omicron XBB subvariants and subsequently JN.1. Boosting was defined as having received at least a third mRNA vaccine dose ≥6 months following completion of an initial two-dose mRNA primary vaccine series.
[d] Including individuals of Indian ethnicity, mixed ethnicity or other ethnicities (eg. Eurasian, Arab); categories consolidated as individual numbers too small for separate analysis.
[e] Housing type was used as an indicator of socioeconomic status.
[f] Comorbidity burden was defined using the Charlson Comorbidity Index (CCMI), which consists of the following comorbidities: myocardial infarction, chronic heart failure, peripheral vascular disease, cerebrovascular accident, dementia, chronic obstructive pulmonary disease, connective tissue disease, peptic ulcer disease, diabetes mellitus, hemiplegia, liver disease, moderate to severe renal impairment, solid tumor, leukemia, human immunodeficiency virus (HIV) infection with AIDS.
[g] Immunocompromised status was defined as: presence of solid malignancy, hematologic malignancy, rheumatologic or inflammatory disorders, organ or stem cell transplant, or other intrinsic immune condition or immunodeficiency.
[h] Pre-existing cardiac history was defined as history of ischemic heart disease or heart failure.

© 2025 Wee LE et al. *JAMA Network Open.*

eTable 5: Prevalence of acute cardiovascular events (composite) amongst hospitalisations for respiratory-syncytial-virus, influenza and Omicron XBB/JN.1 SARS-CoV-2 in Singaporean adults aged≥60 years

	**Any cardiovascular event[a]**				**Any cardiac event[b]**				**Any MACE[c]**			
	**RSV, N(%)**	**Other RVI (reference), N(%), unweighted**	**Other RVI (reference), N(%), weighted[d]**	**Adjusted odds ratio, aOR[e] (95%CI)**	**RSV, N(%)**	**Other RVI (reference), N(%), unweighted**	**Other RVI (reference), N(%), weighted[d]**	**Adjusted odds ratio, aOR[e] (95%CI)**	**RSV, N(%)**	**Other RVI (reference), N(%), unweighted**	**Other RVI (reference), N(%), weighted[d]**	**Adjusted odds ratio, aOR[e] (95%CI)**
**Hospitalisations for respiratory viral infections**												
RSV versus COVID-19 (reference), boosted[f]	204 (12.0%)	1027 (8.6%)	155 (9.3%)	**1.31 (1.11-1.56)**	194 (11.5%)	816 (6.9%)	128 (7.7%)	**1.54 (1.29-1.84)**	98 (5.8%)	606 (5.1%)	89 (5.4%)	1.04 (0.83-1.32)
RSV versus COVID-19 (reference), unboosted[f]	204 (12.0%)	129 (8.1%)	120 (7.6%)	**1.67 (1.29-2.16)**	194 (11.5%)	109 (6.9%)	97 (6.1%)	**2.00 (1.52-2.64)**	98 (5.8%)	74 (4.7%)	62 (3.9%)	**1.48 (1.05-2.08)**
RSV versus unvaccinated influenza (reference)[g]	204 (12.0%)	691 (10.8%)	202 (12.1%)	0.98 (0.82-1.17)	194 (11.5%)	619 (9.7%)	184 (11.0%)	1.03 (0.85-1.24)	98 (5.8%)	347 (5.4%)	106 (6.4%)	0.88 (0.69-1.13)
RSV versus vaccinated influenza (reference)[g]	204 (12.0%)	194 (10.3%)	195 (12.1%)	0.98 (0.78-1.23)	194 (11.5%)	177 (9.4%)	179 (11.1%)	1.02 (0.80-1.29)	98 (5.8%)	89 (4.7%)	91 (5.6%)	1.00 (0.73-1.39)

MACE, major adverse cardiovascular event; OR, odds ratio; CI, confidence interval; RVI, respiratory-viral-infection
Numbers in parentheses refer to 95% confidence intervals.

[a] Any cardiovascular event defined as the composite of any cardiac, cerebrovascular or thrombotic event

[b] Any cardiac event defined as the composite of any dysrhythmia, ischemic heart disease, heart failure, or other cardiac event

[c] Major-adverse-cardiovascular/cerebrovascular-event (MACE) was defined as the first incidence of myocardial infarction, stroke, heart failure, ventricular arrhythmia, or sudden cardiovascular collapse.

[d] Weighted prevalence of cardiovascular events, calculated using propensity-score-weighting for the following covariates: age, gender, ethnicity, socioeconomic status, comorbidity burden (Charlson Comorbidity Index), immunocompromised status, pre-existing cardiac history, pre-existing diabetes, pre-existing dyslipidemia; with RSV hospitalisations as the reference category.

[e] Odds ratio estimated using multivariate logistic regression models, adjusting for age, gender, ethnicity, socioeconomic status, comorbidity burden (Charlson Comorbidity Index), immunocompromised status, pre-existing cardiac history, pre-existing diabetes, pre-existing dyslipidemia, and intensive-care-unit (ICU) admission during hospitalisation for respiratory viral infection. Age was treated as a continuous variable in regression analyses.

[f] RSV hospitalisations were compared against hospitalisations attributed to COVID-19 in January 2023-June 2024 (COVID-19 as the reference category); during 2023-2024, community transmission of COVID-19 was dominated by various Omicron XBB subvariant and subsequently JN.1. Boosting was defined as having received at least a third mRNA vaccine dose ≥6 months following completion of an initial two-dose mRNA primary vaccine series.

[g] RSV hospitalisations were compared against hospitalisations attributed to influenza from January 2017- June 2024 (influenza hospitalisations as the reference category). Individuals who had received seasonal influenza vaccination <365 days prior to hospitalisation were considered vaccinated.

© 2025 Wee LE et al. *JAMA Network Open.*

eTable 6: Prevalence of acute cardiovascular events (individual) amongst hospitalisations for respiratory-syncytial-virus, influenza and Omicron XBB/JN.1 SARS-CoV-2 in Singaporean adults aged≥60 years

Acute cardiovascular events	RSV, acute cardiovascular events, N(%)	Boosted COVID-19, acute cardiovascular events, N(%), unweighted	Boosted COVID-19, acute cardiovascular events, N(%), weighted[b,c]	Adjusted odds ratio, aOR[e] (95%CI), acute cardiovascular events, RSV versus boosted COVID-19 (reference)
Any dysrhythmia	90 (5.3%)	345 (2.9%)	57 (3.4%)	**1.59 (1.24-2.05)**
Any ischemic heart disease	55 (3.2%)	351 (3.0%)	51 (3.0%)	1.02 (0.76-1.38)
Any heart failure	55 (3.2%)	189 (1.6%)	32 (1.9%)	**1.68 (1.22-2.32)**
Any other cardiac event[a]	59 (3.5%)	220 (1.9%)	36 (2.2%)	**1.59 (1.17-2.18)**
Any thrombotic event	4 (0.2%)	58 (0.5%)	8 (0.5%)	0.47 (0.17-1.32)
Any cerebrovascular event	8 (0.5%)	183 (1.5%)	26 (1.6%)	**0.29 (0.14-0.59)**
**Acute cardiovascular events**	**RSV, acute cardiovascular events, N(%)**	**Unboosted COVID-19, acute cardiovascular events, N(%), unweighted**	**Unboosted COVID-19, acute cardiovascular events, N(%), weighted[b,c]**	**Adjusted odds ratio, aOR[e] (95%CI), acute cardiovascular events, RSV versus unboosted COVID-19 (reference)**
Any dysrhythmia	90 (5.3%)	51 (3.2%)	52 (3.2%)	**1.69 (1.14-2.48)**
Any ischemic heart disease	55 (3.2%)	38 (2.4%)	30 (1.9%)	**1.67 (1.05-2.63)**
Any heart failure	55 (3.2%)	27 (1.7%)	20 (1.3%)	**2.58 (1.58-4.22)**
Any other cardiac event[a]	59 (3.5%)	33 (2.1%)	24 (1.5%)	**2.29 (1.43-3.66)**
Any thrombotic event	4 (0.2%)	8 (0.5%)	9 (0.6%)	0.43 (0.12-1.53)
Any cerebrovascular event	8 (0.5%)	14 (0.9%)	16 (1.0%)	0.45 (0.18-1.15)
**Acute cardiovascular events**	**RSV, acute cardiovascular events, N(%)**	**Vaccinated influenza, acute cardiovascular events, N(%), unweighted**	**Vaccinated influenza, acute cardiovascular events, N(%), weighted[b,d]**	**Adjusted odds ratio, aOR[e] (95%CI), acute cardiovascular events, RSV versus vaccinated influenza (reference)**
Any dysrhythmia	90 (5.3%)	89 (4.7%)	97 (6.0%)	0.87 (0.63-1.20)
Any ischemic heart disease	55 (3.2%)	64 (3.4%)	57 (3.5%)	0.89 (0.60-1.32)
Any heart failure	55 (3.2%)	34 (1.8%)	39 (2.4%)	1.33 (0.83-2.15)
Any other cardiac event[a]	59 (3.5%)	39 (2.1%)	43 (2.7%)	1.30 (0.82-2.04)
Any thrombotic event	4 (0.2%)	7 (0.4%)	6 (0.4%)	0.59 (0.16-2.13)
Any cerebrovascular event	8 (0.5%)	11 (0.6%)	13 (0.8%)	0.59 (0.21-1.60)
**Acute cardiovascular events**	**RSV, acute cardiovascular events, N(%)**	**Unvaccinated influenza, acute cardiovascular events, N(%), unweighted**	**Unvaccinated influenza, acute cardiovascular events, N(%), weighted[b,d]**	**Adjusted odds ratio, aOR[e] (95%CI), acute cardiovascular events, RSV versus unvaccinated influenza (reference)**
Any dysrhythmia	90 (5.3%)	297 (4.6%)	90 (5.4%)	0.97 (0.75-1.25)
Any ischemic heart disease	55 (3.2%)	211 (3.3%)	58 (3.5%)	0.91 (0.66-1.26)
Any heart failure	55 (3.2%)	154 (2.4%)	54 (3.2%)	0.99 (0.70-1.38)
Any other cardiac event[a]	59 (3.5%)	160 (2.5%)	55 (3.3%)	1.04 (0.75-1.45)
Any thrombotic event	4 (0.2%)	25 (0.4%)	6 (0.4%)	0.64 (0.21-1.92)
Any cerebrovascular event	8 (0.5%)	56 (0.9%)	14 (0.9%)	0.54 (0.25-1.16)

OR, odds ratio; CI, confidence interval; RVI, respiratory-viral-infection

Numbers in parentheses refer to 95% confidence intervals.

[a] Any other cardiac event included: non-ischemic cardiomyopathy; cardiac arrest; cardiogenic shock; inflammatory heart diseases (myocarditis/pericarditis)

[b] Weighted prevalence of cardiovascular events, calculated using propensity-score-weighting for the following covariates: age, gender, ethnicity, socioeconomic status, comorbidity burden (Charlson Comorbidity Index), immunocompromised status, pre-existing cardiac history, pre-existing diabetes, pre-existing dyslipidemia; with RSV hospitalisations as the reference category.

© 2025 Wee LE et al. *JAMA Network Open.*

[c\] Hospitalisations attributed to respiratory-syncytial-virus, RSV, from January 2017- June 2024 were compared against hospitalisations attributed to COVID-19 in January 2023-June 2024 (COVID-19 as the reference category); during 2023-2024, community transmission of COVID-19 was dominated by various Omicron XBB subvariant and subsequently JN.1. Boosting was defined as having received at least a third mRNA vaccine dose ≥6 months following completion of an initial two-dose mRNA primary vaccine series.
[d] RSV hospitalisations were compared against hospitalisations attributed to influenza from January 2017- June 2024 (influenza hospitalisations as the reference category). Individuals who had received seasonal influenza vaccination <365 days prior to hospitalisation were considered vaccinated.
[e] Odds ratio estimated using multivariate logistic regression models, adjusting for age, gender, ethnicity, socioeconomic status, comorbidity burden (Charlson Comorbidity Index), immunocompromised status, pre-existing cardiac history, pre-existing diabetes, preexisting dyslipidemia, and intensive-care-unit (ICU) admission during hospitalisation for respiratory viral infection. Age was treated as a continuous variable in regression analyses.

© 2025 Wee LE et al. *JAMA Network Open.*

eTable 7: Odds of intensive-care-unit (ICU) admission, stratified by presence of an acute cardiovascular event, during acute hospitalisations for respiratory-syncytial-virus, influenza and Omicron XBB/JN.1 SARS-CoV-2 in Singaporean adults aged≥60 years

**Hospitalisations for respiratory viral infections**	**Any cardiovascular event[a]**	**No cardiovascular event[a] (reference)**	**Adjusted odds ratio, aOR[d] (95%CI)**	**Any cardiac event[b]**	**No cardiac event[b] (reference)**	**Adjusted odds ratio, aOR[d] (95%CI)**	**Any MACE[c]**	**No MACE[c] (reference)**	**Adjusted odds ratio, aOR[d] (95%CI)**	**Any ischemic heart disease event**	**No ischemic heart disease event (reference)**	**Adjusted odds ratio, aOR[d] (95%CI)**	**Any heart failure event**	**No heart failure event**	**Adjusted odds ratio, aOR[d] (95%CI)**
	**ICU, N(%)**	**ICU admission, N(%)**		**ICU admission, N(%)**	**ICU admission, N(%)**		**ICU admission, N(%)**	**ICU admission, N(%)**		**ICU admission, N(%)**	**ICU admission, N(%)**		**ICU admission, N(%)**	**ICU admission, N(%)**	
Respiratory-syncytial-virus (RSV) hospitalisations, 2017-2024	10 (8.0%)	60 (3.8%)	**2.21 (1.07-4.59)**	10 (8.3%)	60 (3.8%)	**2.34 (1.12-4.86)**	6 (9.8%)	64 (3.9%)	**2.82 (1.13-7.03)**	5 (15.6%)	65 (3.9%)	**4.44 (1.57-12.54)**	3 (8.3%)	67 (4.0%)	2.32 (0.66-8.12)
Unvaccinated influenza hospitalisations, 2017-2024[e]	56 (11.3%)	213 (3.6%)	**3.45 (2.32-5.12)**	52 (11.6%)	217 (3.7%)	**3.44 (2.28-5.18)**	39 (15.1%)	230 (3.8%)	**5.15 (3.25-8.14)**	24 (16.4%)	245 (3.9%)	**4.48 (2.54-7.92)**	18 (16.7%)	251 (4.0%)	**4.61 (2.40-8.86)**
Vaccinated influenza hospitalisations, 2017-2024[e]	13 (10.8%)	59 (3.3%)	**3.26 (1.58-6.72)**	13 (11.7%)	59 (3.3%)	**3.56 (1.72-7.38)**	10 (17.2%)	62 (3.4%)	**5.35 (2.40-11.93)**	7 (16.3%)	65 (3.5%)	**5.66 (2.31-13.82)**	2 (11.1%)	70 (3.7%)	1.57 (0.28-8.79)
Omicron XBB/JN.1 COVID-19 hospitalisations, 2023-2024, boosted[f]	89 (10.9%)	204 (1.8%)	**6.38 (4.65-8.76)**	79 (12.2%)	214 (1.9%)	**7.24 (5.17-10.16)**	59 (12.2%)	234 (2.1%)	**6.27 (4.40-8.94)**	45 (16.1%)	248 (2.1%)	**8.45 (5.59-12.79)**	15 (10.6%)	278 (2.4%)	**5.03 (2.72-9.31)**
Omicron XBB/JN.1 COVID-19 hospitalisations, 2023-2024, unboosted[f]	10 (9.9%)	36 (2.4%)	**5.20 (2.10-12.88)**	7 (8.2%)	39 (2.6%)	**2.92 (1.06-8.04)**	9 (15.0%)	37 (2.4%)	**12.66 (5.04-31.79)**	3 (10.0%)	43 (2.8%)	**3.99 (1.05-15.18)**	3 (15.8%)	43 (2.7%)	**10.87 (1.74-68.07)**

MACE, major adverse cardiovascular event; OR, odds ratio; CI, confidence interval

Numbers in parentheses refer to 95% confidence intervals.

[a] Any cardiovascular event defined as the composite of any cardiac, cerebrovascular or thrombotic event

[b] Any cardiac event defined as the composite of any dysrhythmia, ischemic heart disease, heart failure, or other cardiac event

[c] Major-adverse-cardiovascular/cerebrovascular-event (MACE) was defined as the first incidence of myocardial infarction, stroke, heart failure, ventricular arrhythmia, or sudden cardiovascular collapse.

[d] Odds ratio estimated using multivariate logistic regression models, adjusting for age, gender, ethnicity, socioeconomic status, comorbidity burden (Charlson Comorbidity Index), immunocompromised status, pre-existing cardiac history, pre-existing diabetes, preexisting dyslipidemia, and intensive-care-unit (ICU) admission during hospitalisation for respiratory viral infection. Age was treated as a continuous variable in regression analyses.

[e] Individuals who had received seasonal influenza vaccination <365 days prior to hospitalisation were considered vaccinated.

[f] Boosting was defined as having received at least a third mRNA vaccine dose ≥6 months following completion of an initial two-dose mRNA primary vaccine series.

© 2025 Wee LE et al. *JAMA Network Open.*

e-Table 8: Sociodemographic and clinical characteristics of contemporaneous hospitalisations during 2023-2024 for respiratory-syncytial-virus, influenza and Omicron XBB/JN.1 SARS-CoV-2 in Singaporean adults

Sociodemographic and clinical characteristics	Respiratory-syncytial-virus (RSV) hospitalisations, 2023-2024[a] N(%)	Influenza hospitalisations, 2023-2024, vaccinated[b] N(%)	Influenza hospitalisations, 2023-2024, unvaccinated[b] N(%)	Omicron XBB/JN.1 COVID-19 hospitalisations, 2023-2024	
				**Boosted,[c] N(%)**	**Unboosted,[c] N(%)**
Total number of hospitalisations, N(%)	1,371 (5.6%)	1,584 (6.5%)	5,151 (21.0%)	14,485 (59.1%)	1,938 (7.9%)
**Age, years**					
Mean age, years (S.D)	72.8 (15.701)	68.9 (16.985)	60.9 (20.126)	71.8 (16.131)	73.3 (16.934)
<60 years	230 (16.8%)	313 (19.8%)	2,082 (40.4%)	2,595 (17.9%)	354 (18.3%)
60-69 years	249 (18.2%)	292 (18.4%)	958 (18.6%)	2,550 (17.6%)	301 (15.5%)
70-79 years	357 (26.0%)	565 (35.7%)	1,157 (22.5%)	4,083 (28.2%)	453 (23.4%)
≥80 years	535 (39.0%)	414 (26.1%)	954 (18.5%)	5,257 (36.3%)	830 (42.8%)
**Ethnicity**					
Chinese	853 (62.2%)	1,011 (63.8%)	2,932 (56.9%)	11,042 (76.2%)	1,432 (73.9%)
Malay	315 (23.0%)	364 (23.0%)	1,311 (25.5%)	2,095 (14.5%)	247 (12.7%)
Others[d]	203 (14.8%)	209 (13.2%)	908 (17.6%)	1,348 (9.3%)	259 (13.4%)
**Gender**					
Female	796 (58.1%)	769 (48.5%)	2,716 (52.7%)	7,034 (48.6%)	1,013 (52.3%)
**Socioeconomic status (housing type)[e]**					
Public, 1-2 room	160 (11.7%)	176 (11.1%)	627 (12.2%)	1,607 (11.1%)	254 (13.1%)
Public, 3-room	303 (22.1%)	327 (20.6%)	1,074 (20.9%)	3,253 (22.5%)	435 (22.4%)
Public, 4-room	446 (32.5%)	544 (34.3%)	1,645 (31.9%)	4,476 (30.9%)	619 (31.9%)
Public, 5-room	368 (26.8%)	434 (27.4%)	1,530 (29.7%)	3,988 (27.5%)	472 (24.4%)
Private housing	94 (6.9%)	103 (6.5%)	275 (5.3%)	1,161 (8.0%)	158 (8.2%)
**Comorbidity burden[f]**					
Charlson Comorbidity Index, CCMI=0	271 (19.8%)	387 (24.4%)	2,247 (43.6%)	4,010 (27.7%)	492 (25.4%)
Mild (CCMI=1-2)	756 (55.1%)	887 (56.0%)	2,256 (43.8%)	7,425 (51.3%)	944 (48.7%)
Moderate-severe (CCMI≥3)	344 (25.1%)	310 (19.6%)	648 (12.6%)	3,050 (21.1%)	502 (25.9%)
**Immunocompromised[g]**					
Yes	287 (20.9%)	313 (19.8%)	735 (14.3%)	2,715 (18.7%)	424 (21.9%)
**Pre-existing cardiac history[h]**					
Yes	429 (31.3%)	368 (23.2%)	902 (17.5%)	3,328 (23.0%)	566 (29.2%)
**Pre-existing dyslipidemia**					
Yes	111 (8.1%)	111 (7.0%)	261 (5.1%)	883 (6.1%)	132 (6.8%)
**Pre-existing diabetes**					
Yes	581 (42.4%)	664 (41.9%)	1,614 (31.3%)	5,845 (40.4%)	750 (38.7%)
**Pre-existing chronic lung disease**					

© 2025 Wee LE et al. *JAMA Network Open.*

Yes	354 (25.8%)	444 (28.0%)	700 (13.6%)	1,743 (12.0%)	242 (12.5%)
**Clinical outcomes and healthcare utilisation**					
Required intensive-care-unit (ICU) admission	46 (3.4%)	50 (3.2%)	140 (2.7%)	362 (2.5%)	59 (3.0%)
Length-of-stay, days (median, IQR)	5.0 (3.0-8.0)	4.0 (2.0-7.0)	3.0 (2.0-6.0)	4.0 (2.0-8.0)	5.0 (2.0-10.0)
ICU length-of-stay, days (median, IQR)	2.0 (1.0-5.0)	3.0 (1.0-6.0)	2.0 (1.0-4.0)	2.0 (1.0-5.0)	3.0 (1.0-10.0)

[a] Hospitalisations attributed to respiratory-syncytial-virus, RSV, from January 2023- June 2024.
[b] Hospitalisations attributed to influenza from January 2023- June 2024. Individuals who had received seasonal influenza vaccination <365 days prior to hospitalisation were considered vaccinated.
[c] Hospitalisations attributed to COVID-19 in January 2023-June 2024; during 2023-2024, community transmission of COVID-19 was dominated by various Omicron XBB subvariants and subsequently JN.1. Boosting was defined as having received at least a third mRNA vaccine dose ≥6 months following completion of an initial two-dose mRNA primary vaccine series.
[d] Including individuals of Indian ethnicity, mixed ethnicity or other ethnicities (eg. Eurasian, Arab); categories consolidated as individual numbers too small for separate analysis.
[e] Housing type was used as an indicator of socioeconomic status.
[f] Comorbidity burden was defined using the Charlson Comorbidity Index (CCMI), which consists of the following comorbidities: myocardial infarction, chronic heart failure, peripheral vascular disease, cerebrovascular accident, dementia, chronic obstructive pulmonary disease, connective tissue disease, peptic ulcer disease, diabetes mellitus, hemiplegia, liver disease, moderate to severe renal impairment, solid tumor, leukemia, human immunodeficiency virus (HIV) infection with AIDS.
[g] Immunocompromised status was defined as: presence of solid malignancy, hematologic malignancy, rheumatologic or inflammatory disorders, organ or stem cell transplant, or other intrinsic immune condition or immunodeficiency.
[h] Pre-existing cardiac history was defined as history of ischemic heart disease or heart failure.

© 2025 Wee LE et al. *JAMA Network Open.*

eTable 9: Prevalence of acute cardiovascular events (composite) amongst contemporaneous hospitalisations during 2023-2024 for respiratory-syncytial-virus, influenza and Omicron XBB/JN.1 SARS-CoV-2 in Singaporean adults

	**Any cardiovascular event[a]**				**Any cardiac event[b]**				**Any MACE[c]**			
	**RSV, N(%)**	**Other RVI (reference), N(%), unweighted**	**Other RVI (reference), N(%), weighted[d]**	**Adjusted odds ratio, aOR[e] (95%CI)**	**RSV, N(%)**	**Other RVI (reference), N(%), unweighted**	**Other RVI (reference), N(%), weighted[d]**	**Adjusted odds ratio, aOR[e] (95%CI)**	**RSV, N(%)**	**Other RVI (reference), N(%), unweighted**	**Other RVI (reference), N(%), weighted[d]**	**Adjusted odds ratio, aOR[e] (95%CI)**
**Hospitalisations for respiratory viral infections**												
RSV versus COVID-19 (reference), boosted[f]	167 (12.2%)	1180 (8.1%)	123 (9.1%)	**1.39 (1.15-1.66)**	158 (11.5%)	941 (6.5%)	103 (7.6%)	**1.59 (1.32-1.93)**	76 (5.5%)	681 (4.7%)	70 (5.2%)	1.05 (0.81-1.36)
RSV versus COVID-19 (reference), unboosted[f]	167 (12.2%)	158 (8.2%)	99 (7.8%)	**1.66 (1.29-2.15)**	158 (11.5%)	131 (6.8%)	78 (6.2%)	**2.01 (1.53-2.64)**	76 (5.5%)	92 (4.7%)	55 (4.3%)	1.29 (0.91-1.83)
RSV versus unvaccinated influenza (reference)[g]	167 (12.2%)	464 (9.0%)	149 (11.2%)	1.10 (0.89-1.36)	158 (11.5%)	414 (8.0%)	135 (10.2%)	1.16 (0.93-1.44)	76 (5.5%)	219 (4.3%)	84 (6.3%)	0.86 (0.64-1.16)
RSV versus vaccinated influenza (reference)[g]	167 (12.2%)	148 (9.3%)	136 (10.7%)	1.16 (0.90-1.50)	158 (11.5%)	132 (8.3%)	121 (9.5%)	1.24 (0.95-1.63)	76 (5.5%)	59 (3.7%)	56 (4.4%)	1.28 (0.87-1.87)

MACE, major adverse cardiovascular event; OR, odds ratio; CI, confidence interval; RVI, respiratory-viral-infection
Numbers in parentheses refer to 95% confidence intervals.

[a] Any cardiovascular event defined as the composite of any cardiac, cerebrovascular or thrombotic event

[b] Any cardiac event defined as the composite of any dysrhythmia, ischemic heart disease, heart failure, or other cardiac event

[c] Major-adverse-cardiovascular/cerebrovascular-event (MACE) was defined as the first incidence of myocardial infarction, stroke, heart failure, ventricular arrhythmia, or sudden cardiovascular collapse.

[d] Weighted prevalence of cardiovascular events, calculated using propensity-score-weighting for the following covariates: age, gender, ethnicity, socioeconomic status, comorbidity burden (Charlson Comorbidity Index), immunocompromised status, pre-existing cardiac history, pre-existing diabetes, pre-existing dyslipidemia; with RSV hospitalisations as the reference category.

[e] Odds ratio estimated using multivariate logistic regression models, adjusting for age, gender, ethnicity, socioeconomic status, comorbidity burden (Charlson Comorbidity Index), immunocompromised status, pre-existing cardiac history, pre-existing diabetes, pre-existing dyslipidemia, and intensive-care-unit (ICU) admission during hospitalisation for respiratory viral infection. Age was treated as a continuous variable in regression analyses.

[f] RSV hospitalisations were compared against contemporaneous hospitalisations attributed to COVID-19 in January 2023-June 2024 (COVID-19 as the reference category); during 2023-2024, community transmission of COVID-19 was dominated by various Omicron XBB subvariant and subsequently JN.1. Boosting was defined as having received at least a third mRNA vaccine dose ≥6 months following completion of an initial two-dose mRNA primary vaccine series.

[g] RSV hospitalisations were compared against contemporaneous hospitalisations attributed to influenza from January 2023- June 2024 (influenza hospitalisations as the reference category). Individuals who had received seasonal influenza vaccination <365 days prior to hospitalisation were considered vaccinated.

© 2025 Wee LE et al. *JAMA Network Open.*

eTable 10: Prevalence of acute cardiovascular events (individual) amongst contemporaneous hospitalisations during 2023-2024 for respiratory-syncytial-virus, influenza and Omicron XBB/JN.1 SARS-CoV-2 in Singaporean adults

Acute cardiovascular events	RSV, acute cardiovascular events, N(%)	Boosted COVID-19, acute cardiovascular events, N(%), unweighted	Boosted COVID-19, acute cardiovascular events, N(%), weighted[b,c]	Adjusted odds ratio, aOR[e] (95%CI), acute cardiovascular events, RSV versus boosted COVID-19 (reference)
Any dysrhythmia	77 (5.6%)	396 (2.7%)	46 (3.4%)	**1.71 (1.31-2.23)**
Any ischemic heart disease	35 (2.6%)	405 (2.8%)	41 (3.0%)	0.82 (0.57-1.17)
Any heart failure	47 (3.4%)	223 (1.5%)	25 (1.9%)	**1.85 (1.32-2.59)**
Any other cardiac event[a]	53 (3.9%)	262 (1.8%)	29 (2.2%)	**1.81 (1.32-2.49)**
Any thrombotic event	5 (0.4%)	73 (0.5%)	6 (0.5%)	0.74 (0.29-1.86)
Any cerebrovascular event	6 (0.4%)	199 (1.4%)	20 (1.5%)	**0.28 (0.12-0.65)**
**Acute cardiovascular events**	**RSV, acute cardiovascular events, N(%)**	**Unboosted COVID-19, acute cardiovascular events, N(%), unweighted**	**Unboosted COVID-19, acute cardiovascular events, N(%), weighted[b,c]**	**Adjusted odds ratio, aOR[e] (95%CI), acute cardiovascular events, RSV versus unboosted COVID-19 (reference)**
Any dysrhythmia	77 (5.6%)	61 (3.1%)	41 (3.3%)	**1.78 (1.21-2.62)**
Any ischemic heart disease	35 (2.6%)	48 (2.5%)	27 (2.1%)	1.20 (0.75-1.93)
Any heart failure	47 (3.4%)	30 (1.5%)	16 (1.2%)	**2.89 (1.77-4.72)**
Any other cardiac event[a]	53 (3.9%)	38 (2.0%)	19 (1.5%)	**2.69 (1.70-4.25)**
Any thrombotic event	5 (0.4%)	12 (0.6%)	11 (0.9%)	0.40 (0.13-1.23)
Any cerebrovascular event	6 (0.4%)	19 (1.0%)	14 (1.1%)	0.39 (0.15-1.05)
**Acute cardiovascular events**	**RSV, acute cardiovascular events, N(%)**	**Vaccinated influenza, acute cardiovascular events, N(%), unweighted**	**Vaccinated influenza, acute cardiovascular events, N(%), weighted[b,d]**	**Adjusted odds ratio, aOR[e] (95%CI), acute cardiovascular events, RSV versus vaccinated influenza (reference)**
Any dysrhythmia	77 (5.6%)	75 (4.7%)	68 (5.4%)	1.04 (0.73-1.49)
Any ischemic heart disease	35 (2.6%)	39 (2.5%)	35 (2.8%)	0.92 (0.56-1.50)
Any heart failure	47 (3.4%)	22 (1.4%)	21 (1.7%)	**2.09 (1.21-3.59)**
Any other cardiac event[a]	53 (3.9%)	28 (1.8%)	26 (2.1%)	**1.94 (1.18-3.19)**
Any thrombotic event	5 (0.4%)	8 (0.5%)	7 (0.6%)	0.65 (0.21-2.05)
Any cerebrovascular event	6 (0.4%)	8 (0.5%)	8 (0.6%)	0.68 (0.22-2.08)
**Acute cardiovascular events**	**RSV, acute cardiovascular events, N(%)**	**Unvaccinated influenza, acute cardiovascular events, N(%), unweighted**	**Unvaccinated influenza, acute cardiovascular events, N(%), weighted[b,d]**	**Adjusted odds ratio, aOR[e] (95%CI), acute cardiovascular events, RSV versus unvaccinated influenza (reference)**
Any dysrhythmia	77 (5.6%)	221 (4.3%)	63 (4.7%)	1.20 (0.90-1.61)
Any ischemic heart disease	35 (2.6%)	121 (2.3%)	43 (3.2%)	0.79 (0.52-1.19)
Any heart failure	47 (3.4%)	92 (1.8%)	44 (3.3%)	1.05 (0.71-1.57)
Any other cardiac event[a]	53 (3.9%)	101 (2.0%)	45 (3.4%)	1.15 (0.78-1.69)
Any thrombotic event	5 (0.4%)	11 (0.2%)	3 (0.2%)	1.46 (0.47-4.57)
Any cerebrovascular event	6 (0.4%)	42 (0.8%)	12 (0.9%)	0.49 (0.20-1.19)

© 2025 Wee LE et al. *JAMA Network Open.*

OR, odds ratio; CI, confidence interval; RVI, respiratory-viral-infection
Numbers in parentheses refer to 95% confidence intervals.

[a] Any other cardiac event included: non-ischemic cardiomyopathy; cardiac arrest; cardiogenic shock; inflammatory heart diseases (myocarditis/pericarditis)

[b] Weighted prevalence of cardiovascular events, calculated using propensity-score-weighting for the following covariates: age, gender, ethnicity, socioeconomic status, comorbidity burden (Charlson Comorbidity Index), immunocompromised status, pre-existing cardiac history, pre-existing diabetes, pre-existing dyslipidemia; with RSV hospitalisations as the reference category.

[c\] Hospitalisations attributed to respiratory-syncytial-virus, RSV, from January 2017- June 2024 were compared against hospitalisations attributed to COVID-19 in January 2023-June 2024 (COVID-19 as the reference category); during 2023-2024, community transmission of COVID-19 was dominated by various Omicron XBB subvariant and subsequently JN.1. Boosting was defined as having received at least a third mRNA vaccine dose ≥6 months following completion of an initial two-dose mRNA primary vaccine series.

[d] RSV hospitalisations were compared against hospitalisations attributed to influenza from January 2017- June 2024 (influenza hospitalisations as the reference category). Vaccination for seasonal influenza was defined as receiving a dose of influenza vaccine within 365 days prior to influenza hospitalisations; unvaccinated individuals had either not received any influenza vaccination dose, or had received influenza vaccination >365 days from influenza hospitalisation. Individuals who had received seasonal influenza vaccination <365 days prior to hospitalisation were considered vaccinated.

[e] Odds ratio estimated using multivariate logistic regression models, adjusting for age, gender, ethnicity, socioeconomic status, comorbidity burden (Charlson Comorbidity Index), immunocompromised status, pre-existing cardiac history, pre-existing diabetes, preexisting dyslipidemia, and intensive-care-unit (ICU) admission during hospitalisation for respiratory viral infection. Age was treated as a continuous variable in regression analyses.

© 2025 Wee LE et al. *JAMA Network Open.*

eTable 11: Odds of intensive-care-unit (ICU) admission, stratified by presence of an acute cardiovascular event, amongst contemporaneous acute hospitalisations during 2023-2024 for respiratory-syncytial-virus, influenza and Omicron XBB/JN.1 SARS-CoV-2 in Singaporean adults

Hospitalisations for respiratory viral infections	Any cardiovascular event[a]	No cardiovascular event[a] (reference)	Adjusted odds ratio, aOR[d] (95%CI)	Any cardiac event[b]	No cardiac event[b] (reference)	Adjusted odds ratio, aOR[d] (95%CI)	Any MACE[c]	No MACE[c] (reference)	Adjusted odds ratio, aOR[d] (95%CI)	Any ischemic heart disease event	No ischemic heart disease event (reference)	Adjusted odds ratio, aOR[d] (95%CI)	Any heart failure event	No heart failure event	Adjusted odds ratio, aOR[d] (95%CI)
	ICU, N(%)	ICU admission, N(%)		ICU admission, N(%)	ICU admission, N(%)		ICU admission, N(%)	ICU admission, N(%)		ICU admission, N(%)	ICU admission, N(%)		ICU admission, N(%)	ICU admission, N(%)	
Respiratory-syncytial-virus (RSV) hospitalisations, 2023-2024	7 (6.7%)	39 (3.1%)	**2.45 (1.01-5.94)**	7 (7.1%)	39 (3.1%)	**2.65 (1.09-6.45)**	4 (7.4%)	42 (3.2%)	2.54 (0.83-7.72)	3 (14.3%)	43 (3.2%)	**5.27 (1.48-18.79)**	2 (5.9%)	44 (3.3%)	2.05 (0.46-9.03)
Unvaccinated influenza hospitalisations, 2017-2024[e]	21 (6.4%)	119 (2.5%)	**2.21 (1.22-4.01)**	17 (5.8%)	123 (2.5%)	1.86 (0.97-3.57)	13 (8.8%)	127 (2.5%)	**3.04 (1.48-6.22)**	7 (8.9%)	133 (2.6%)	**2.86 (1.12-7.29)**	4 (6.6%)	136 (2.7%)	1.56 (0.44-5.50)
Vaccinated influenza hospitalisations, 2017-2024[e]	7 (7.3%)	43 (2.9%)	**3.29 (1.33-8.14)**	7 (8.0%)	43 (2.9%)	**3.78 (1.53-9.36)**	4 (9.5%)	46 (3.0%)	**4.01 (1.33-12.14)**	2 (7.1%)	48 (3.1%)	2.84 (0.56-14.31)	1 (7.7%)	49 (3.1%)	2.64 (0.29-24.18)
Omicron XBB/JN.1 COVID-19 hospitalisations, 2023-2024, boosted[f]	106 (11.3%)	256 (1.9%)	**6.52 (4.89-8.70)**	94 (12.6%)	268 (2.0%)	**7.21 (5.29-9.81)**	72 (13.3%)	290 (2.1%)	**7.43 (5.36-10.29)**	53 (16.5%)	309 (2.2%)	**7.27 (4.95-10.66)**	20 (12.0%)	342 (2.4%)	**5.54 (3.17-9.67)**
Omicron XBB/JN.1 COVID-19 hospitalisations, 2023-2024, unboosted[f]	15 (12.0%)	44 (2.4%)	**6.77 (3.20-14.33)**	12 (11.7%)	47 (2.6%)	**4.86 (2.16-10.93)**	13 (17.8%)	46 (2.5%)	**13.84 (6.27-30.53)**	5 (13.5%)	54 (2.8%)	**3.88 (1.21-12.42)**	3 (15.0%)	56 (2.9%)	**7.51 (1.51-37.24)**

MACE, major adverse cardiovascular event; OR, odds ratio; CI, confidence interval
Numbers in parentheses refer to 95% confidence intervals.
[a] Any cardiovascular event defined as the composite of any cardiac, cerebrovascular or thrombotic event
[b] Any cardiac event defined as the composite of any dysrhythmia, ischemic heart disease, heart failure, or other cardiac event
[c] Major-adverse-cardiovascular/cerebrovascular-event (MACE) was defined as the first incidence of myocardial infarction, stroke, heart failure, ventricular arrhythmia, or sudden cardiovascular collapse.
[d] Odds ratio estimated using multivariate logistic regression models, adjusting for age, gender, ethnicity, socioeconomic status, comorbidity burden (Charlson Comorbidity Index), immunocompromised status, pre-existing cardiac history, pre-existing diabetes, preexisting dyslipidemia, and intensive-care-unit (ICU) admission during hospitalisation for respiratory viral infection. Age was treated as a continuous variable in regression analyses.
[e] Individuals who had received seasonal influenza vaccination <365 days prior to hospitalisation were considered vaccinated.
[f] Boosting was defined as having received at least a third mRNA vaccine dose ≥6 months following completion of an initial two-dose mRNA primary vaccine series.

© 2025 Wee LE et al. *JAMA Network Open.*

eTable 12: Prevalence of acute cardiovascular events (composite) amongst hospitalisations for respiratory-syncytial-virus versus PCR- confirmed Omicron XBB/JN.1 SARS-CoV-2 in Singaporean adults

	**Any cardiovascular event[a]**				**Any cardiac event[b]**				**Any MACE[c]**			
	**RSV, N(%)**	**Other RVI (reference), N(%), unweighted**	**Other RVI (reference), N(%), weighted[d]**	**Adjusted odds ratio, aOR[e] (95%CI)**	**RSV, N(%)**	**Other RVI (reference), N(%), unweighted**	**Other RVI (reference), N(%), weighted[d]**	**Adjusted odds ratio, aOR[e] (95%CI)**	**RSV, N(%)**	**Other RVI (reference), N(%), unweighted**	**Other RVI (reference), N(%), weighted[d]**	**Adjusted odds ratio, aOR[e] (95%CI)**
**Hospitalisations for respiratory viral infections**												
RSV versus PCR-confirmed COVID-19 (reference), boosted[f]	234 (10.9%)	850 (9.0%)	182 (8.6%)	**1.29 (1.09-1.52)**	220 (10.2%)	679 (7.2%)	147 (7.0%)	**1.52 (1.28-1.81)**	113 (5.3%)	489 (5.2%)	101 (4.8%)	1.07 (0.85-1.34)
RSV versus PCR-confirmed COVID-19 (reference), unboosted[f]	234 (10.9%)	126 (9.0%)	146 (7.4%)	**1.55 (1.19-2.02)**	220 (10.2%)	104 (7.4%)	116 (5.8%)	**1.89 (1.42-2.51)**	113 (5.3%)	72 (5.2%)	82 (4.1%)	1.27 (0.90-1.80)

MACE, major adverse cardiovascular event; OR, odds ratio; CI, confidence interval; RVI, respiratory-viral-infection
Numbers in parentheses refer to 95% confidence intervals.

[a] Any cardiovascular event defined as the composite of any cardiac, cerebrovascular or thrombotic event

[b] Any cardiac event defined as the composite of any dysrhythmia, ischemic heart disease, heart failure, or other cardiac event

[c] Major-adverse-cardiovascular/cerebrovascular-event (MACE) was defined as the first incidence of myocardial infarction, stroke, heart failure, ventricular arrhythmia, or sudden cardiovascular collapse.

[d] Weighted prevalence of cardiovascular events, calculated using propensity-score-weighting for the following covariates: age, gender, ethnicity, socioeconomic status, comorbidity burden (Charlson Comorbidity Index), immunocompromised status, pre-existing cardiac history, pre-existing diabetes, pre-existing dyslipidemia; with RSV hospitalisations as the reference category.

[e] Odds ratio estimated using multivariate logistic regression models, adjusting for age, gender, ethnicity, socioeconomic status, comorbidity burden (Charlson Comorbidity Index), immunocompromised status, pre-existing cardiac history, pre-existing diabetes, pre-existing dyslipidemia, and intensive-care-unit (ICU) admission during hospitalisation for respiratory viral infection. Age was treated as a continuous variable in regression analyses.

[f] RSV hospitalisations from January 2017-June 2024 were compared against hospitalisations attributed to COVID-19 in January 2023-June 2024 (COVID-19 as the reference category) confirmed via positive PCR test (antigen-positive cases without corresponding confirmatory PCR were excluded). During 2023-2024, community transmission of COVID-19 was dominated by various Omicron XBB subvariant and subsequently JN.1. Boosting was defined as having received at least a third mRNA vaccine dose ≥6 months following completion of an initial two-dose mRNA primary vaccine series.

© 2025 Wee LE et al. *JAMA Network Open.*

eTable 13: Prevalence of acute cardiovascular events (individual) amongst hospitalisations for respiratory-syncytial-virus versus PCR-confirmed Omicron XBB/JN.1 SARS-CoV-2 in Singaporean adults

Acute cardiovascular events	RSV, acute cardiovascular events, N(%)	Boosted COVID-19, acute cardiovascular events, N(%), unweighted	Boosted COVID-19, acute cardiovascular events, N(%), weighted[c,d]	Adjusted odds ratio, aOR[f] (95%CI), acute cardiovascular events, RSV versus boosted COVID-19 (reference)
Any dysrhythmia[a]	99 (4.6%)	286 (3.0%)	62 (3.0%)	**1.59 (1.24-2.05)**
Any ischemic heart disease	61 (2.8%)	297 (3.2%)	61 (2.9%)	0.94 (0.70-1.26)
Any heart failure	66 (3.1%)	156 (1.7%)	37 (1.8%)	**1.75 (1.28-2.40)**
Any other cardiac event[b]	72 (3.4%)	188 (2.0%)	43 (2.1%)	**1.62 (1.20-2.19)**
Any thrombotic event	7 (0.3%)	59 (0.6%)	12 (0.6%)	0.55 (0.24-1.23)
Any cerebrovascular event	9 (0.4%)	139 (1.5%)	29 (1.4%)	**0.30 (0.15-0.60)**
**Acute cardiovascular events**	**RSV, acute cardiovascular events, N(%)**	**Unboosted COVID-19, acute cardiovascular events, N(%), unweighted**	**Unboosted COVID-19, acute cardiovascular events, N(%), weighted[c,d]**	**Adjusted odds ratio, aOR[f] (95%CI), acute cardiovascular events, RSV versus unboosted COVID-19 (reference)**
Any dysrhythmia[a]	99 (4.6%)	50 (3.6%)	62 (3.1%)	**1.54 (1.03-2.30)**
Any ischemic heart disease	61 (2.8%)	35 (2.5%)	35 (1.7%)	1.62 (1.01-2.61)
Any heart failure	66 (3.1%)	25 (1.8%)	23 (1.1%)	**2.77 (1.60-4.79)**
Any other cardiac event[b]	72 (3.4%)	33 (2.4%)	33 (1.7%)	**2.05 (1.26-3.32)**
Any thrombotic event	7 (0.3%)	11 (0.8%)	20 (1.0%)	**0.31 (0.11-0.86)**
Any cerebrovascular event	9 (0.4%)	15 (1.1%)	20 (1.0%)	0.42 (0.18-1.02)

OR, odds ratio; CI, confidence interval; RVI, respiratory-viral-infection. Numbers in parentheses refer to 95% confidence intervals.

[a] Any other cardiac event included: non-ischemic cardiomyopathy; cardiac arrest; cardiogenic shock; inflammatory heart diseases (myocarditis/pericarditis)

[b] Weighted prevalence of cardiovascular events, calculated using propensity-score-weighting for the following covariates: age, gender, ethnicity, socioeconomic status, comorbidity burden (Charlson Comorbidity Index), immunocompromised status, pre-existing cardiac history, pre-existing diabetes, pre-existing dyslipidemia; with RSV hospitalisations as the reference category.

[c] Odds ratio estimated using multivariate logistic regression models, adjusting for age, gender, ethnicity, socioeconomic status, comorbidity burden (Charlson Comorbidity Index), immunocompromised status, pre-existing cardiac history, pre-existing diabetes, preexisting dyslipidemia, and intensive-care-unit (ICU) admission during hospitalisation for respiratory viral infection. Age was treated as a continuous variable in regression analyses.

[d\] Hospitalisations attributed to respiratory-syncytial-virus, RSV, from January 2017- June 2024 were compared against contemporaneous hospitalisations attributed to COVID-19 in January 2023-June 2024 confirmed via positive PCR test (antigen-positive cases without corresponding confirmatory PCR were excluded). COVID-19 hospitalisations were used as the reference category. During 2023-2024, community transmission of COVID-19 was dominated by various Omicron XBB subvariant and subsequently JN.1. Boosting was defined as having received at least a third mRNA vaccine dose ≥6 months following completion of an initial two-dose mRNA primary vaccine series.

© 2025 Wee LE et al. *JAMA Network Open.*

eAppendix 1: List of ICD-10 codes used for outcomes of interest

RSV/influenza hospitalizations, ICD-10 codes

**ICD-10 codes**	**Description**
**Influenza hospitalizations**[a]	
J10.0[x]	Influenza due to other identified influenza virus with pneumonia
J10.1[x]	Influenza due to other identified influenza virus with other respiratory manifestations
J10.8[x]	Influenza due to other identified influenza virus with other manifestations
**RSV hospitalizations**[b]	
J12.1	Respiratory syncytial virus pneumonia
J20.5	Acute bronchitis due to respiratory syncytial virus
J21.0	Acute bronchiolitis due to respiratory syncytial virus
B97.4	Respiratory syncytial virus as the cause of diseases classified elsewhere

[a]For influenza hospitalizations, ICD-10 codes specific for influenza (J10.0[x], J10.1[x], J10.8[x]) were utilised to identify influenza hospitalizations in Mediclaims data; [x] denotes that all other subcodes of the parent ICD-10 code were included (eg. for J10.0[x], all other subcodes, including J10.00, influenza due to other identified influenza virus with unspecified type of pneumonia; J10.01, influenza due to other identified influenza virus with the same other identified influenza virus pneumonia; J10.08, influenza due to other identified influenza virus with other specified pneumonia, were additionally included.

[b] For RSV hospitalizations, RSV-specific ICD-10 codes were used (J12.1, J20.5, J21.0, B97.4).

COVID-19 hospitalizations

For COVID-19 hospitalizations, as the corresponding ICD-10 code (U07.1) was not in use for discharge coding during the study period, data on COVID-19 hospitalizations was obtained from the national registry maintained by the local Ministry-of-Health (MOH), which contained details of all COVID-19 hospitalisations as well as all COVID-19 test results (both polymerase-chain-reaction testing [PCR], and rapid-antigen-tests [RAT]). While both COVID-19 test results (positive PCR/RAT) and hospitalizations attributable to COVID-19 were legally notifiable to the MOH under the Infectious Diseases Act, Singapore, only hospitalizations notified by healthcare providers as attributable to COVID-19 were included for a basis of comparison. The top 10 ICD-10 diagnosis codes for notified COVID-19 hospitalizations are given in the table below, covering 60.1% (9874/16423) of notified COVID-19 hospitalizations:

© 2025 Wee LE et al. *JAMA Network Open.*

Order (highest to lowest)	ICD-10 codes	Description	Proportion of COVID-19 hospitalizations, N(%)
1	J06.9	Acute upper respiratory infection, unspecified	2469 (15.0)
2	J12.89	Other viral pneumonia	1880 (11.4)
3	B97.29	Coronavirus as the cause of diseases classified elsewhere	1689 (10.3)
4	J18.9	Pneumonia, unspecified	1157 (7.0)
5	B33.8	Other specified viral diseases	987 (6.0)
6	J22[x]	Unspecified acute lower respiratory infection	523 (3.2)
7	B34.2	Coronavirus infection, unspecified site	380 (2.3)
8	I50[x]	Congestive heart failure	323 (2.0)
9	J45[x]	Asthma	293 (1.8)
10	A41.9	Sepsis, unspecified	173 (1.1)

[x] denotes that all other subcodes of the parent ICD-10 code were included

Comorbidities (Charlson Comorbidity Index, CCMI)

Comorbidity burden was defined at the point of hospital admission using the Charlson Comorbidity Index (CCMI), which consists of the following comorbidities: myocardial infarction, chronic heart failure, peripheral vascular disease, cerebrovascular accident, dementia, chronic obstructive pulmonary disease, connective tissue disease, peptic ulcer disease, diabetes mellitus, hemiplegia, liver disease, moderate to severe renal impairment, solid tumor, leukemia, human immunodeficiency virus (HIV) infection with AIDS. CCMI was computed based on ICD-10 codes recorded in Mediclaims data using a look-back period of 4 years prior to hospitalization.

Immunocompromised status

Immunocompromised status was defined as: presence of solid malignancy, hematologic malignancy, rheumatologic or inflammatory disorders, organ or stem cell transplant, or other intrinsic immune condition or immunodeficiency, at the point of hospital admission, using ICD-10 codes recorded in Mediclaims data and a look-back period of 4 years prior to hospitalization. Diagnosis codes included: solid malignancy (ICD-10 codes: C00–C80, C7A, C7B, D3A, Z51.0, and Z51.1), hematologic malignancy (ICD-10 codes: C81–C86, C88, C90–C96, D46, D61.0, D70.0, D61.2, D61.9, and D71), rheumatologic or inflammatory disorder (ICD-10 codes: D86, E85 [except E85.0], G35, J67.9, L40.54, L40.59, L93.0, L93.2, L94, M05–M08, M30, M31.3, M31.5, M32–M34, M35.3, M35.8, M35.9, M46, and T78.40), other intrinsic immune condition or immunodeficiency (ICD-10 codes: D27.9, D61.09, D72.89, D80, D81 [except D81.3], D82–D84, D89 [except D89.2], K70.3, K70.4, K72, K74.3–K74.6 [except K74.60 and K74.69], N04, and R18), or organ or stem cell transplant (ICD-10 codes: T86 [except T86.82–T86.84, T86.89, and T86.9], D47.Z1, Z48.2, Z94, and Z98.85).

© 2025 Wee LE et al. *JAMA Network Open.*

List of ICD-10 codes used for outcomes of interest (cardiovascular events)

Outcome	ICD 10 Code	Description
**Dysrhythmia**		
Atrial fibrillation	I48	Atrial fibrillation and flutter
Sinus tachycardia	I47	Paroxysmal tachycardia
	R00.0	Tachycardia, unspecified
Sinus bradycardia	R00.1	Bradycardia, unspecified
Other arrhythmias	I44	Atrioventricular and left bundle-branch block
	R00.2	Palpitations
	R00.8	Other abnormalities of heart beat
	R00.9	Unspecified abnormalities of heart beat
	I45	Other conduction disorders
	I49	Other cardiac arrhythmias
**Acute myocardial infarction**		
Myocardial infarction	I21	Acute myocardial infarction
	I22	Subsequent ST elevation (STEMI) and non-ST elevation (NSTEMI) myocardial infarction
Acute coronary disease	I24	Other acute ischemic heart diseases
	I25.10	Atherosclerotic heart disease of native coronary artery without angina pectoris
	I25.11	Atherosclerotic heart disease of native coronary artery with angina pectoris
Ischemic cardiomyopathy	I25.5	Ischemic cardiomyopathy
Angina	I20	Angina pectoris
**Heart failure**		
Heart failure	I50	Heart failure
	I50.21	Acute systolic (congestive) heart failure
	150.23	Acute on chronic systolic (congestive) heart failure
	150.31	Acute diastolic (congestive) heart failure
	I50.33	Acute on chronic diastolic (congestive) heart failure
	I50.41	Acute combined systolic (congestive) and diastolic (congestive) heart failure
	I50.43	Acute on chronic combined systolic (congestive) and diastolic (congestive) heart failure
	I50.811	Acute right heart failure
	I50.813	Acute on chronic right heart failure
**Other cardiac event**		
Non-ischemic cardiomyopathy	I42	Cardiomyopathy
Cardiac arrest	I46	Cardiac arrest
Cardiogenic shock	R57.0	Cardiogenic shock
**Thrombotic events**		
Pulmonary embolism	I26	Pulmonary embolism

© 2025 Wee LE et al. *JAMA Network Open.*

Deep venous thrombosis	I80.1	Phlebitis and thrombophlebitis of femoral vein
	I80.2	Phlebitis and thrombophlebitis of other and unspecified deep vessels of lower extremities
	I80.8	Phlebitis and thrombophlebitis of other sites
	I81	Portal vein thrombosis
	I82	Other venous embolism and thrombosis
	I67.6	Nonpyogenic thrombosis of intracranial venous system
**Cerebrovascular event**		
Ischemic stroke	I63	Cerebral infarction
	G46	Vascular syndromes of brain in cerebrovascular diseases
Transient ischemic attack (TIA)	G45	Transient cerebral ischaemic attacks and related syndromes
	I65	Occlusion and stenosis of precerebral arteries not resulting in cerebral infarction
	I66	Occlusion and stenosis of cerebral arteries not resulting in cerebral infarction
Haemorrhagic stroke	I60	Nontraumatic subarachnoid hemorrhage
	I61	Intracerebral hemorrhage
	I62	Other non-traumatic intracranial hemorrhage

© 2025 Wee LE et al. *JAMA Network Open.*
