# Supplementary material for: Cardiac Events in Adults Hospitalized for Respiratory Syncytial Virus vs COVID-19 or Influenza
Source: JAMA Netw Open. 2025 May 22;8(5):e2511764. doi: 10.1001/jamanetworkopen.2025.11764 (PMC12100453; doi:10.1001/jamanetworkopen.2025.11764)
Supplement: Supplement 2. — Data Sharing Statement [file jamanetwopen-e2511764-s002.pdf]

Data Sharing Statement

Wee. Cardiac Events in Adults Hospitalized for Respiratory Syncytial Virus vs COVID-19 or Influenza. *JAMA Netw Open*. Published May 21, 2025.
doi:10.1001/jamanetworkopen.2025.11764

Data
Data available: No

Additional Information
Explanation for why data not available: Individual patient data is not available due to data protection requirements. Aggregated anonymised data can be made available, subject to approval by the Ministry of Health, Singapore. Requests to be made to the corresponding author.
